# Increased Expression of YAP Inhibited the Autophagy Level by Upregulating mTOR Signal in the Eutopic ESCs of Endometriosis

**DOI:** 10.3389/fendo.2022.813165

**Published:** 2022-01-31

**Authors:** Tianjiao Pei, Bin Luo, Wei Huang, Dong Liu, Yujing Li, Li Xiao, Xin Huang, Yunwei Ouyang, Huili Zhu

**Affiliations:** ^1^Department of Reproductive Medicine, West China Second University Hospital of Sichuan University, Chengdu, China; ^2^Key Laboratory of Birth Defects and Related Diseases of Women and Children (Sichuan University), Ministry of Education, West China Second University Hospital of Sichuan University, Chengdu, China; ^3^Department of Reproductive Endocrinology, West China Second University Hospital of Sichuan University, Chengdu, China

**Keywords:** endometriosis, Yes-associated protein (YAP), autophagy, eutopic endometrial stromal cells (eutopic ESCs), mammalian target of rapamycin (mTOR), decidualization

## Abstract

We first reported that the Hippo-YAP signaling pathway plays a critical role in the pathogenesis of endometriosis (EMS). Autophagy is also related to the invasion ability of endometrial cells and is involved in the pathogenesis of EMS through multi-levels. However, the precise regulatory mechanism of YAP on autophagy in the eutopic endometrial stromal cells (ESCs) is still unclear. Primary eutopic ESCs of EMS patients (*n* = 12) and control patients without EMS (*n* = 9) were isolated and cultured to investigate the expressions of YAP and mTOR, the role of YAP in autophagy, and the effect of the YAP-autophagy signal on the decidualization of the eutopic ESCs. Endometriosis-related sequencing data (GSE51981) in the GEO database were used to find the genes significantly correlated with YAP. We found 155 genes with significant differences in the interaction with YAP in EMS from the dataset, and the autophagy pathway was significantly enriched. Following on from our previous studies of YAP knockdown, overexpression of YAP resulted in an increased expression of mTOR and decreased ratio of LC3-II/LC3-I and autophagy markers, in the eutopic ESCs; transmission electron microscope observation also showed fewer autophagosomes compared with the control cells. Furthermore, ESCs of the Rapamycin-treated group showed significant decidual-like changes with significantly increased decidual prolactin level at 72 h after *in vitro* decidualization. These results demonstrate that the increased YAP inhibited the level of autophagy by upregulating the mTOR signal in the eutopic ESCs of endometriosis. The YAP-autophagy signal plays an important role in the pathogenesis of endometriosis-associated infertility.

## Introduction

Endometriosis (EMS) is a chronic inflammatory hormone-dependent disease, characterized by the growth of the endometrium outside the uterus, affecting more than 190 million women worldwide and up to 10% of women of reproductive age (1–3). Chronic pelvic pain and infertility caused by the disease seriously affect women’s reproductive health and quality of life. According to statistics, about 25%–50% of infertile women suffer from EMS, while 30%–50% of patients with EMS suffer from infertility (4–6). Before a definitive diagnosis is made, women often endure symptoms for years with negative effects on wellbeing and quality of life (3). EMS is invasive and recurrent, so effective and thorough treatment and reduction of disease recurrence have become one of the most urgent and difficult problems in clinical practice.

The influence of EMS on women’s fertility is mainly related to the abnormalities of pelvic anatomical structure, changes of abdominal microenvironment, ovarian function abnormalities, and endometrial receptivity abnormalities (7). The receptivity of endometrium is one of the important factors affecting women’s fecundity. The endometrium is a complex tissue with periodic changes, which is regulated by ovarian steroids, autocrine/paracrine, and signaling pathways. During the secretion period, endometrial stromal cells undergo decidualization under the action of estrogens, which plays a crucial role in the establishment and maintenance of pregnancy. At present, endometrial receptivity abnormalities in EMS are considered to include defects in the proliferative phase, reduction of integrin αvβ3 and its direct transcriptional regulator HOXA10, and progesterone resistance (8–10). In addition, many studies have found that some signaling pathways play an important role in endometriosis, such as the over-activated MAPK pathway and PI3K/AKT pathway, which affect the role of progesterone and block the decidualization of endometrial stromal cells (ESCs) (11). In a word, abnormal regulation of endometrial signaling pathways, local inflammation, stromal differentiation, and improper endometrial reconstruction in EMS may lead to a condition of endometrium that is unacceptable for implanting embryos (8, 12).

As one of the core effector components of the Hippo signaling pathway, Yes-associated protein (YAP) is a molecule closely related to organ formation and malignancy. When the phosphorylation of YAP occurs because of intracellular and extracellular signals mediated by upstream regulatory molecules and core molecules, the phosphorylated YAP protein accumulates in the cytoplasm or degrades through the ubiquitination pathway, at which time the regulatory function of the Hippo pathway is inhibited, whereas when YAP protein is not phosphorylated, it will enter the nucleus and bind to TEA domain transcription factor, jointly regulating the expression of downstream target genes, cell proliferation, migration, and survival (13). It is currently believed that the Hippo pathway can integrate the functions of multiple signaling pathways to form a complex signaling network. YAP and its downstream transcription factors determine cell behavior in a coordinated manner and play an important role in organ development, tumor genesis and development, epithelial–mesenchymal transformation, and other cell biological behaviors (14). Abnormal programmed cell apoptosis and reduced apoptotic susceptibility play a key role in the development and invasion of EMS. Our group first reported in 2016 (15) that YAP knockdown in the eutopic ESCs decreased cell proliferation and enhanced cell apoptosis, while overexpression of YAP resulted in increased proliferation and decreased apoptosis of ESCs. We also found that after treatment with Verteporfin in the EMS animal model of nude mice, the size of endometriotic lesions was significantly reduced. It hints that the Hippo-YAP signaling pathway plays a critical role in the pathogenesis of EMS.

Autophagy, as a highly effective subcellular degradation pathway, can remove chromosomes with gene mutation damage and abnormal structure and aging or damaged organelles, carry out subcellular level reconstruction of cells, provide energy for cells, maintain intracellular material anabolism, and maintain homeostasis of the intracellular environment. In the menstrual cycle, spontaneous and periodic apoptosis of normal endometrium is an important factor to maintain its normal structure and function, and autophagy plays a key regulatory role in the apoptosis of endometrium cells in different phases of the human endometrium cycle (16).

In recent years, basic studies on EMS have shown that abnormal regulation of signaling pathways plays an important role in the occurrence and development of EMS (17). The mammalian target of rapamycin (mTOR) in the PI3K/AKT/mTOR pathway is a serine/threonine protein kinase, which is also the confluence point of the upstream pathway to regulate cell growth, proliferation, movement, survival, etc. Currently, it has been clarified that mTOR is a negative regulator of autophagy and participates in the regulatory mechanism of autophagy. Oncology studies have found that mTOR, as the junction point of the signal pathway, regulates the phosphorylation of YAP so that phosphorylated YAP remains in the cytoplasm and cannot bind to TEAD in the nucleus, thus participating in the regulation of cell metabolism and autophagy (18). Other studies have reported that MST1/2 (Hippo key enzyme) maintains autophagy through autophagy marker protein (LC3), suggesting that there may be a precise dialogue between Hippo signal and autophagy (19).

Many studies have shown that autophagy achieves the regulation of EMS through the interference of multiple pathways at multiple levels, and promotes the occurrence, development, and invasion of EMS (20–22). In 2015, Zhang et al. (15) found that autophagy gene Beclin-1 mRNA and protein expression in ESCs in EMS diseases decreased and were negatively correlated with CA125 level and pain. Choi et al. (23) reported that autophagy and apoptosis are simultaneously involved in the pathological process of EMS, and this process is mediated by mTOR, and the abnormal mTOR activity affects the change of autophagy activity. Rat model studies have shown that there is an autophagy downregulation in both the eutopic and ectopic of endometrium, and the autophagy flow inhibitor hydroxychloroquine (HCQ) can effectively shrink and destroy the ectopic lesions, which is expected to be a new target for the treatment of EMS (21). Recently, we reported that increased expression of YAP is associated with decreased cell autophagy in the eutopic ESCs of EMS (24). Although there is a downregulation of autophagy in EMS, the precise regulation of autophagy in endometrial stromal cells in EMS remains unclear, which may involve multiple signaling pathways.

In recent years, studies on the interaction and regulation of YAP and mTOR/autophagy have been one of the hotspots in the study of tumor cell mechanisms. In 2019, it was reported (25) that YAP is highly expressed in ovarian cancer, and silencing YAP may significantly inhibit the malignant behavior of ovarian cancer cells by regulating the PI3K/Akt/mTOR pathway. Zhou et al. (26) found that YAP promoted multi-drug resistance of liver cancer cells and inhibited autophagy-related cell death. However, there have been no reports on the regulatory relationship between YAP and autophagy, the role of YAP and autophagy in the pathogenesis of EMS, and their effects on endometrial receptivity. Therefore, the objective of this study is to explore the role of YAP in the regulation of cell autophagy in the eutopic ESCs from a subset of women with endometriosis and to understand the effect of the YAP-autophagy signal on the decidualization of the eutopic ESCs.

## Materials and Methods

### Participants

This study was approved by the Ethics Committee of West China Second University Hospital of Sichuan University. Written informed consent was obtained from each patient. All participants aged 20–35 years, with regular menstrual cycles and no history of hormonal treatment for at least 3 months before surgery, were included in the study between September 2017 and August 2018. Those who suffered from infertility associated with factors of fallopian, tube, ovary or uterine, or abnormal semen were excluded from the study. Endometrial samples were collected during hysteroscopy and determined by endometrial pathological dating to be in the mid-secretory phase. Twelve women were laparoscopically diagnosed with endometriosis. Another 9 women with hysteroscopic normal uterine cavity and who were laparoscopically endometriosis-free were treated as controls. Samples of the eutopic endometrium that showed endometrial lesions through pathological examinations were excluded. All samples were immediately transferred to the laboratory for primary cell culture or stored in nitrogen for further analysis.

### Data Collection

The gene expression profile of GSE51981 was downloaded from the Gene Expression Omnibus (GEO) database (https://www.ncbi.nlm.nih.gov/geo/). We selected 111 samples from the GSE51981 dataset, containing a total of 77 endometriosis samples (different menstrual cycle phases and different stages of disease) and 34 non-endometriosis samples without uterine pelvic pathology (27). To find gene sets and pathways significantly correlated with YAP in this research and to understand the signal pathways and functional modules related to YAP in EMS, we used the processed data to filter differentially expressed genes (DEGs) and conducted gene enrichment analysis.

### Differentially Expressed Genes

Gene differential analysis was conducted using the limma R package between EMS samples and non-EMS/normal uterus samples. We considered DEGs as |log2fold change (FC)| > 1 and adjusted *p*-value < 0.05. Heatmap and Volcano Plot were generated by pheatmap package and ggplot package, respectively.

### PPI Network Construction

Protein–protein interaction network analysis provides much valuable information for researchers to understand cell function and biological processes. Many studies have shown that neighboring proteins always have some common characteristics in PPI networks (28). We used Search Tool for the Retrieval of Interacting Genes Database (STRING) [https://www.string-db.org/, (9606.protein.links.v9.1) (29)] to assess PPI information (30). To explore the possible protein interaction and relationship between DEGs and YAP in EMS, we used the STRING analysis and converted the results visually by using Cytoscape software. Protein–protein interaction score > 0.4 was set as significant (30).

### GO Term and KEGG Pathway Enrichment Analyses of DEGs

To explore the signaling pathway and biological function most closely related to YAP in EMS, we conducted GO functional annotation analysis and KEGG pathway enrichment analysis on the DEGs with the most significant interaction with YAP obtained from the previous PPI analysis. KEGG is a comprehensive database that integrates information on genome, chemistry, and system functions. GO is a comprehensive database describing the function of genes, which can be divided into three categories: biological process and cellular component molecular function. The pathway enrichment analysis and functional enrichment analysis of DEGs were analyzed and visualized by Clusterprofiler R package. The standard setting of KEGG pathway enrichment with statistical significance was *p* < 0.05 and enrichment score >2.0. The criteria with statistical significance for GO functional annotation were *p* < 0.05 and enrichment score >1.0.

### Immunofluorescence

When ESCs were passaged to the third passage and cell fusion to 90%, cell suspension after passage was added into the 24-well plate according to the cell density of 50% per well and incubated overnight in an incubator (37°C, 5% CO_2_). The next day, ESCs on chamber slides were washed two times with PBS and fixed in 4% paraformaldehyde for 10 min and then cells were washed with PBS again two times. Cells were permeabilized with 0.2% Triton X-100 (Sigma) for 30 min at room temperature when individual cells and cell clusters were observed under the microscope. After blocking with 5% BSA for 15 min, slides were incubated overnight at 4°C with monoclonal rabbit antihuman YAP (1:200, ab52771, Abcam) and monoclonal rabbit antihuman mTOR (1:300, CST#2983S, Abcam). Primary antibodies were detected by incubation with corresponding IgG secondary antibodies conjugated with Alexa Fluor 594 (red) and 488 (green) (1:500, Invitrogen, Eugene, Oregon, USA) for 1 h. Nuclei of the cells were counterstained with 6-diamino-2-phenylindole (DAPI; Sigma-Aldrich, USA).

### Cell Culture and Transfection

The ESCs were isolated from the eutopic endometrium of the endometriosis and control group, cultured in Dulbecco’s modified Eagle’s medium (DMEM)/F12 (1:1) supplemented with 10% fetal bovine serum (life technologies) and 100 U/ml penicillin/streptomycin at 37°C under a 5% CO_2_ condition. Construction and production of YAP-overexpression (OE) plasmid was made by Shandong Vigene Biosciences Co., Ltd. When cells reached 80% confluence, they were digested, seeded at 1 × 10^5^ cells per six-well plate, cultured to 30% to 40% confluency, and then transfected with the YAP-OE plasmid (1 μg) and empty plasmid using Lipofectamine 3000 transfection reagent (Invitrogen, Carlsbad, CA) according to the manufacturer’s protocol. After transfection for 72 h, cells were harvested for further analysis. The transfection efficiency of YAP was measured by qPCR and Western blotting.

### RNA Isolation, cDNA Synthesis, and Real-Time PCR

Total RNA was isolated from all ESCs using the TRIzol (Life Technologies, Carlsbad, CA) reagent according to the manufacturer’s protocol. RNA quantification and purification were performed using a NanoVue Plus spectrophotometer (Healthcare Bio-Science AB, Uppsala, Sweden). The nucleotide:protein ratios (A260:A280) of all the samples were within the range 1.9–2.1. cDNA was synthesized using a PrimeScript RT reagent kit (Takara Biomedical Technology Co., Ltd., Beijing, China) and was diluted 8-fold for PCR amplification. Amplification and detection *via* qPCR were performed in a total reaction volume of 10 μl, consisting of diluted cDNA (3 μl), SYBR Green real-time PCR Master Mix (Applied Biosystems, Carlsbad, CA) (5 μl), forward primer (1 μl), and reverse primer (1 μl), using a CFX96 Realtime PCR system (Bio-Rad Laboratories), and glyceraldehyde 3-phosphate dehydrogenase (GAPDH) was used as an internal control. The PCR cycle was 20 s at 95°C, then 40 cycles of 10 s at 95°C and 20 s at 60°C. The specificity of PCR products was confirmed by analysis of the dissociation curve. Relative gene expression was calculated using the 2^−ΔΔ^CT method. The sequence-specific primers used to amplify the gene products are shown in **Table 1**. Samples were examined in triplicate, and all experiments were repeated three times.

**Table 1:** Primer sequences used in quantitative real-time PCR.

Gene	The sequence-specific primers
YAP1	Forward primer: 5′-CACAGCATGTTCGAGCTCAT-3′
	Reverse primer: 5′-GATGCTGAGCTGTGGGTGTA-3′
GAPDH	Forward primer: 5′-TGCACCACCAACTGCTTAGC-3′
	Reverse primer: 5′-GGCATGGACTGTGGTCATGAG-3′

### Protein Extraction and Western Blotting

Total cellular protein was isolated from ESCs using RIPA buffer with 1% PMSF (Beyotime Biotechnology, Shanghai, China) and protease inhibitors on ice, and protein concentration was determined using the BCA protein assay kit (Beyotime, Biotechnology, Shanghai, China). Equal amounts of protein extracts (50–100 mg) were separated through 6%, 8%, and 15% polyacrylamide gels containing sodium dodecyl sulfate polyacrylamide gel electrophoresis (SDS-PAGE), respectively, and transferred to 0.45 μm polyvinylidene difluoride (PVDF) membranes (Bio-Rad Laboratories Inc., Hercules, CA, USA). Membranes were blocked in 5% milk for 1 h at room temperature and then were washed and incubated with primary antibodies: rabbit monoclonal anti-YAP (Abcam, USA; 1:1,000 dilution), rabbit monoclonal anti- mTOR (CST, USA; 1:500 dilution), rabbit polyclonal anti-LC-3B (Abcam, USA; 1:500 dilution), rabbit anti-GAPDH (Sigma-Aldrich, USA; 1:3,000 dilution), and rabbit anti-beta Actin (Abcam, USA; 1:1,000 dilution) (**Table 2**). Following the overnight incubation, membranes were washed thrice with 0.1% Tween in Tris-buffered saline (TBST) and incubated with a DyLight 800-conjugated goat anti-rabbit IgG secondary antibodies (Thermo Scientific, USA; 1:10,000 dilution) for 2 h. Protein bands were imaged on an infrared imaging system using a Double color infrared laser imaging system (Odyssey, LI-COR, USA), and quantified by Quantity One software (Bio-Rad Laboratories). Protein levels were normalized to that of the internal control GAPDH and β-actin.

**Table 2:** Antibodies used in this study.

Peptide/Protein Target	Antibody Name	Manufacturer, Catalog No.	Species Raised in; Monoclonal or Polyclonal	Dilution Used	RRID
YAP1 Human	YAP1 antibody	Abcam, #ab52771	Rabbit; monoclonal Rabbit;	1:1,000	AB_2219141
mTOR Human, Mouse, Rat,	mTOR (7C10) Rabbit mAb antibody	CST, 2983S	monoclonal	1:500	AB_2105622
LC-3B Mouse, Human	LC3B antibody	Abcam, #ab51520	Rabbit; polyclonal	1:500	AB_881429
GAPDH Rat, Mouse, Human,	GAPDH antibody	Sigma-Aldrich, #G9545	Rabbit; polyclonal	1:3,000	AB_796208
Rabbit IgG	Goat anti-rabbit IgG secondary antibodies	Thermo Fisher Scientific, SA5-10036	Goat; polyclonal	1:10,000	AB_2556616

### Transmission Electron Microscope Observation

To observe the autophagy and ultrastructural changes of the eutopic ESCs after YAP-OE transfection, we performed a TEM observation. Transfected cells were digested and centrifuged, the supernatant was discarded, the precipitate was kept, and fixed in 3% glutaraldehyde. After washing in 0.1 M phosphate buffer, the samples were postfixed with 1% osmium tetroxide in the same buffer for 1 h at 4 °C. Then, the samples were dehydrated with a series of the graded acetone solution, and the samples were next embedded in Epon. Ultrathin sections (~50 nm) were obtained by an ultramicrotome (Leica Ultracut UCT, Germany). Ultrathin sections were double stained with uranyl acetate and lead citrate, and they were examined in a TEM (JEM-1400PLUS, Japan) to detect autophagosomes.

### *In Vitro* Decidualization

To explore the effect of YAP-mTOR signal on the decidualization of the eutopic ESCs, we performed an *in vitro* decidualization induction of the eutopic ESCs after interfering with the YAP function by YAP-TEAD inhibitor Verteporfin (S1786, Selleckchem, USA) (1 μM, 18 h) and blocking the mTOR signal by Rapamycin (S1039, Selleckchem, USA) (100 nM, 4 h) which also can induce autophagy. Verteporfin and Rapamycin treatments were described previously (24). DMSO is the negative control group. Decidualization was induced in ESCs at 70% to 80% confluence after Verteporfin and Rapamycin treatments in 10% charcoal-stripped phenol red-free medium for 24 h. The medium was then replaced, and the cells were cultured with 2% charcoal-stripped phenol red-free medium supplemented with 0.5 mM 8-Br-cAMP (Abcam, Cambridge, MA) and 1 mM medroxyprogesterone acetate (MPA) (Sigma-Aldrich, St. Louis, MO) for 72 h. The culture medium was collected every 24 h, and the supernatants were incubated at −20°C to detect decidual prolactin (dPRL). dPRL protein levels in the supernatants, which are a representative marker of decidual cells, were determined using a commercially available ELISA kit (Cusabio Biotech, Wuhan, China). The ESCs were observed under an inverted microscope every 24 h to study their morphological changes.

### Statistical Analysis

All statistical analyses were performed using the software program SAS version 9.2 (SAS, Institute Inc, USA). The normally distributed data were analyzed by Student’s *t*-test. Data were represented as the mean ± standard deviation (SD). *p* < 0.05 was considered statistically significant (two-tailed).

## Results

### Screening of Differentially Expressed Genes

**Figure 1:**
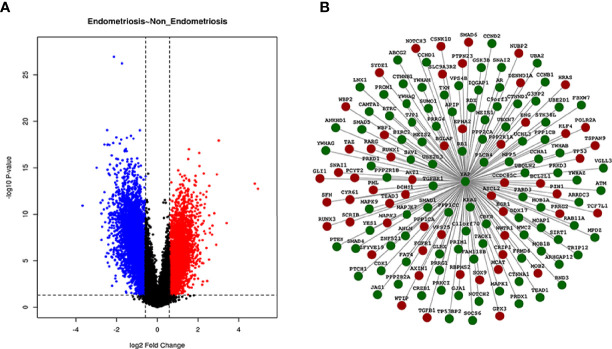
Volcano Plot and PPI network of DEGs. In the Volcano Plot, genes that are upregulated are in red, those that are downregulated are in blue, and those that are insignificantly different are in black **(A)**. Different protein interaction graph with YAP interaction scores greater than 0.4 in GSE51981 samples were shown in the PPI network. Green indicates downregulated expression and red indicates upregulated expression **(B)**.

The R package “limma” was used to screen DEGs between EMS and non-endometriosis samples without uterine pelvic pathology in GSE51981, where a total of 829 DEGs associated with YAP were screened under the threshold of |log2fold change (FC)| > 1 and adjusted *p*-value <0.05. All 829 DEGs are listed in **Supplementary Table S1**. The Volcano Plot is shown in **Figure 1A**. Genes that are upregulated are in red, those that are downregulated are in blue, and those that are insignificantly different are in black.

### PPI Network Integration

We used the STRING database to find the gene sets significantly correlated with YAP in EMS. A total of 155 DEGs with significant differences in the interaction with YAP in EMS were found from the GSE51981 chip samples through the analysis of the protein interaction network (protein–protein interaction score > 0.4 was set as significant). The heatmap of these 155 DEGs with significant differences in the interaction with YAP is shown in **Supplementary Figure S1**, and DEGs are listed in **Supplementary Table S2**. A PPI network of DEGs was performed as shown in **Figure 1B**.

### GO Biological Process Analysis and KEGG Pathway Enrichment of DEGs

**Figure 2:**
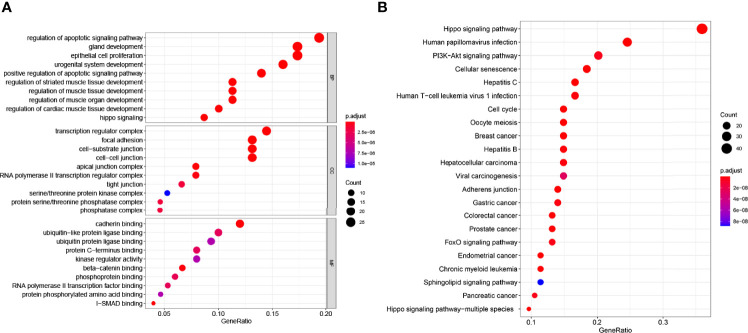
GO and KEGG enrichment analysis of the 155 genes significantly associated with YAP. **(A)** The abscissa is the percentage of genes under the GO functional module (ratio); the ordinate is the GO functional module: BP (biological process), CC (cell composition), and MF (molecular function). **(B)** The *x-*coordinate in the figure is the number of DEGs annotated to KEGG pathway/the total number of differentially expressed genes (ratio); the ordinate is the KEGG pathway. The size of the point represents the number of DEGs annotated to the KEGG pathway.

GO analysis of genes includes biological processes (BP), cell composition (CC), and molecular function (MF). In our study, GO analysis was used to perform the functional process of the DEGs with significant differences in the interaction with YAP in EMS. A *p*-value <0.05 and enrichment score >1.0 were defined to identify regulated genes in GO functional enrichments. The results are shown in **Figure 2A**. GO biological process analysis found that DEGs were mainly enriched in the regulation of apoptotic signaling pathway, gland development, epithelial cell proliferation, urogenital system development, and positive regulation of apoptotic signaling pathway. In the cell composition part, the DEGs were involved in the transcription regulator complex, focal adhesion, cell–substrate junction, and cell–cell junction. In the molecular function section, the genes participated in cadherin binding, ubiquitin-like protein ligase binding, ubiquitin protein ligase binding, and protein C-terminus binding. These results suggested that DEGs with significant differences in the interaction with YAP were mostly involved in cell pathways, cell cycle, cell junction, and binding. All GO analysis is listed in **Supplementary Table S3**.

We also performed KEGG pathway analysis. A *p*-value <0.05 and enrichment fold >2.0 were defined to identify regulated genes in KEGG pathway analysis enrichments. The results are shown in **Figure 2B**. Among these enriched signaling pathways, the Hippo signaling pathway was the most significantly associated with YAP interaction DEGs, while other enriched signaling pathways included cell cycle and division-related pathways (cellular senescence, cell cycle, and oocyte meiosis), liver disease-associated pathways (hepatocellular carcinoma, hepatitis C, and hepatitis B), multiple cancer-related pathways (breast cancer, gastric cancer, colorectal cancer, prostate cancer, and endometrial cancer), and the PI3K-Akt signaling pathway, which is closely associated with tumor immunity. It is worth noting that DEGs with significant differences in the interaction with YAP in EMS are enriched in the autophagy signaling pathway (*p*<0.05) (**Supplementary Table S4**). It suggests that YAP may be correlated with autophagy in EMS.

### The Protein Locations of YAP and mTOR in the Eutopic ESCs

**Figure 3:**
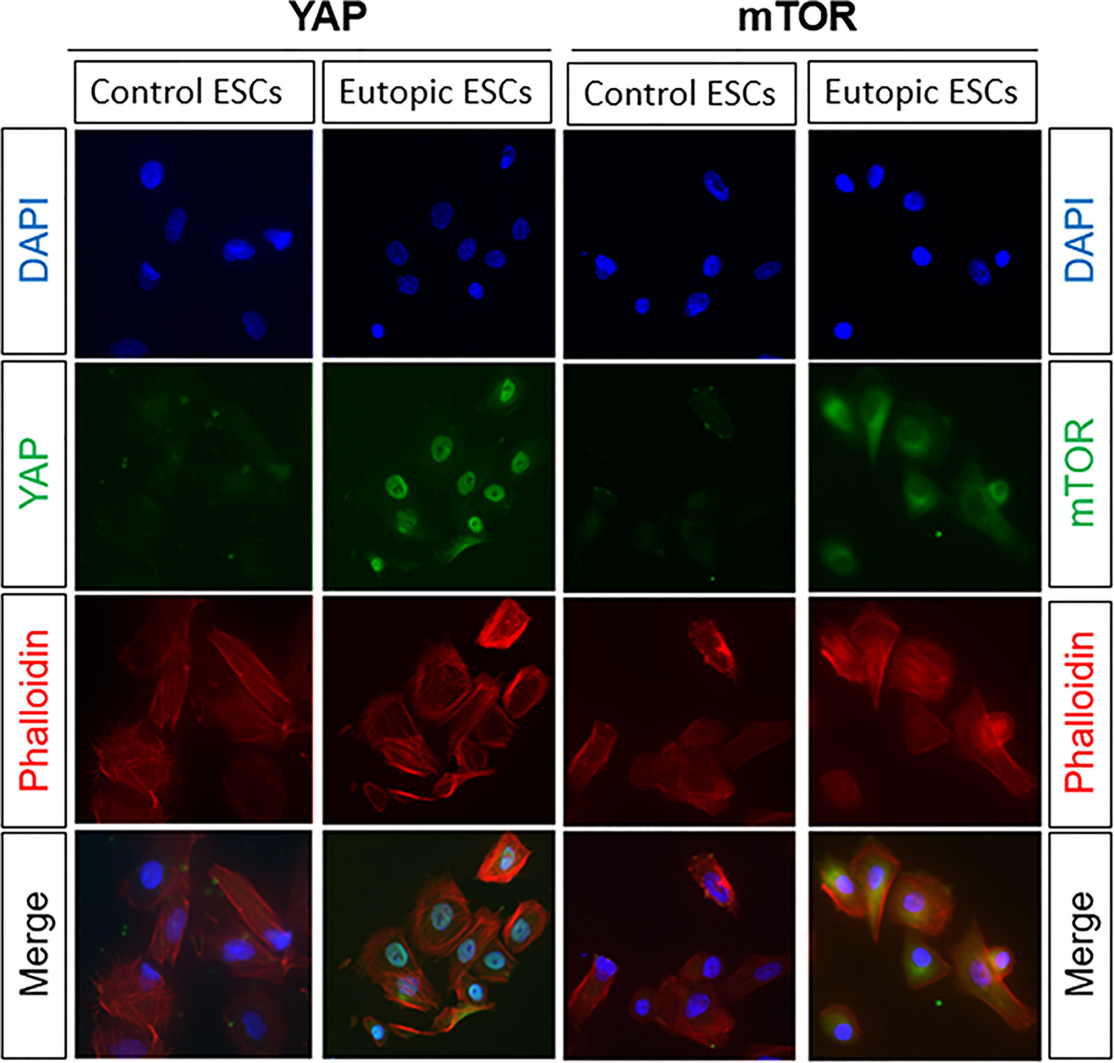
The protein locations of YAP and mTOR in the eutopic ESCs. The results of immunofluorescence showed that YAP was mainly expressed in the nucleus of the eutopic ESCs and a little in the cytoplasm, whereas in the normal ESCs, YAP was mainly located in the cytoplasm and slightly expressed in the nucleus. mTOR was mainly expressed in the cytoplasm of the eutopic ESCs and a little in the nucleus, whereas mTOR was expressed in small amounts in the cytoplasm of normal ESCs, but it was hardly expressed in the nucleus.

Our previous study detected the mRNA and protein levels of YAP and mTOR, negative regulator of autophagy, in the eutopic ESCs. To explore the protein locations of YAP and mTOR in the eutopic ESCs, we performed immunofluorescence. The results showed that YAP was mainly expressed in the nucleus of the eutopic ESCs and a little in the cytoplasm, whereas in the normal ESCs, YAP was mainly located in the cytoplasm and slightly expressed in the nucleus (**Figure 3**). mTOR was mainly expressed in the cytoplasm of the eutopic ESCs and a little in the nucleus, whereas mTOR was expressed in small amounts in the cytoplasm of normal ESCs, but it was hardly expressed in the nucleus (**Figure 3**).

### Overexpression of YAP in the Eutopic ESCs of Endometriosis Inhibited Autophagy Level

**Figure 4:**
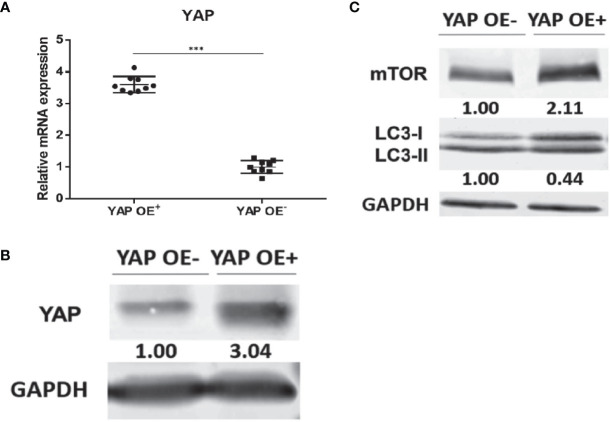
Overexpression of YAP in the eutopic ESCs of endometriosis inhibited autophagy level. The qPCR showed that the expression of YAP mRNA was increased significantly (3.60 ± 0.16 vs. 1.00 ± 0.21; *P* = 0.0006) after transfection with YAP-overexpression plasmid in the eutopic ESCs compared with controls (the eutopic ESCs transfected with the empty plasmid) **(A)**. Western blotting revealed that we obtained a high OE efficiency at the YAP protein level (3.04 vs. 1.00) **(B)**. The expression of mTOR protein (2.11 vs. 1.00) was significantly increased in the eutopic ESCs compared with controls following YAP-OE. By contrast, there was a significantly decreased ratio of the autophagy marker protein LC3-II/LC3-I (0.44 vs. 1.00) **(C)**. ***p = 0.0006.

**Figure 5:**
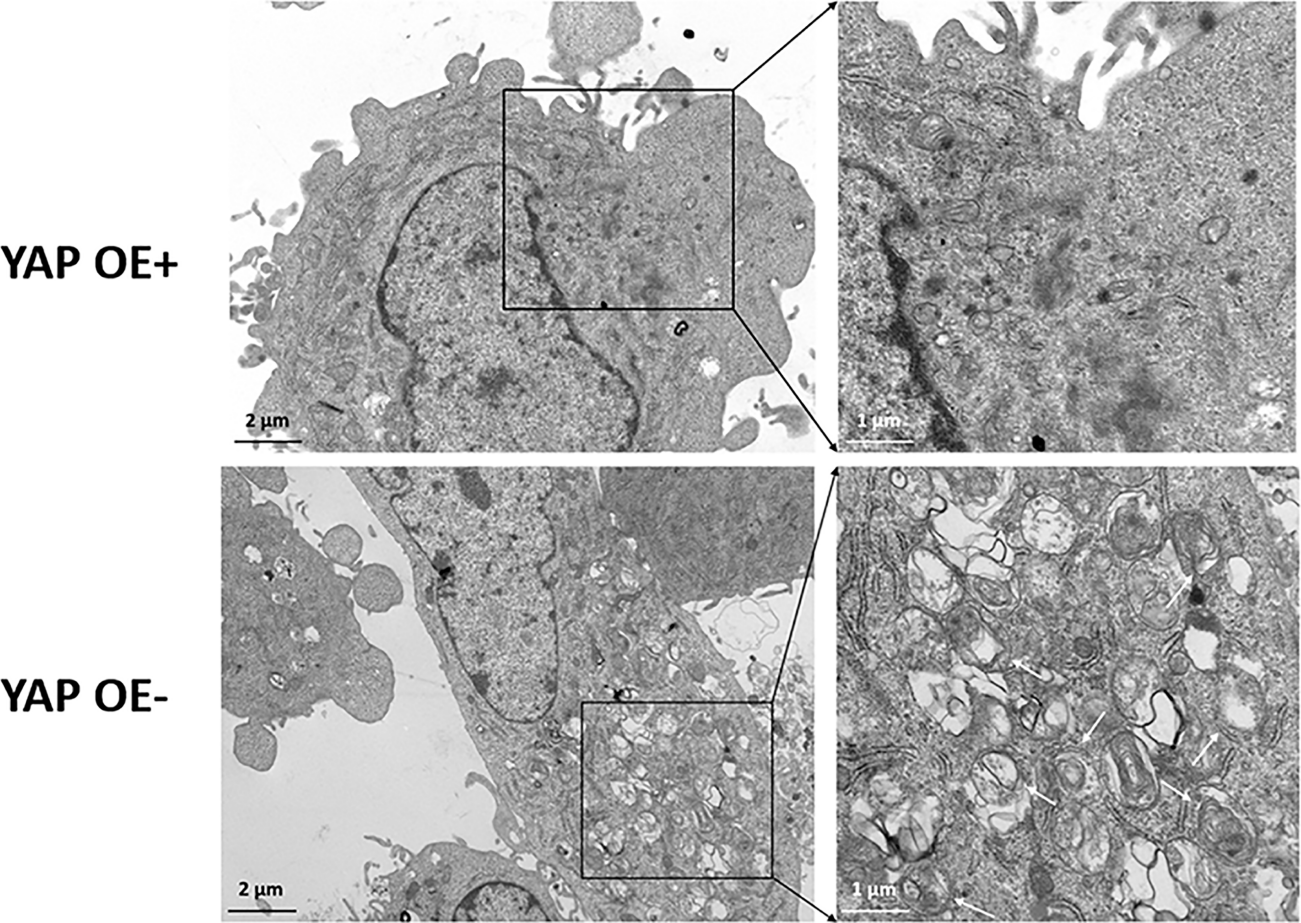
Transmission electron microscope (TEM) observation of autophagosomes after overexpression of YAP in the eutopic ESCs of endometriosis. TEM observation showed the fewer autophagosomes in the YAP-OE group compared with the control cells.

Based on our previous finding that knockdown of YAP in the eutopic ESCs of EMS increases autophagy level, we supplemented the YAP-OE experiment to further explore and clarify the regulatory mechanism of YAP on cell autophagy. The eutopic ESCs were transfected with a YAP-OE plasmid. The empty plasmid was used as control. qPCR showed that the expression of YAP mRNA was increased significantly (3.60 ± 0.16 vs. 1.00 ± 0.21; *p* = 0.0006) after transfection with YAP-OE plasmid in the eutopic ESCs compared with controls (**Figure 4A**). Western blotting revealed that we obtained a high OE efficiency at the YAP protein level (3.04 vs. 1.00) (**Figure 4B**). It combined to suggest that the overexpression of YAP worked. The expression of mTOR protein (2.11 vs. 1.00) was significantly increased in the eutopic ESCs compared with controls following YAP-OE. By contrast, there was a significantly decreased ratio of the autophagy marker protein LC3-II/LC3-I (0.44 vs. 1.00) (**Figure 4C**). TEM observation also showed fewer autophagosomes in the YAP-OE group compared with the control cells (**Figure 5**). These data demonstrated that overexpression of YAP in the eutopic ESCs of endometriosis inhibited the level of cell autophagy.

### Rapamycin Promotes the *In Vitro* Decidualization of ESCs

**Figure 6:**
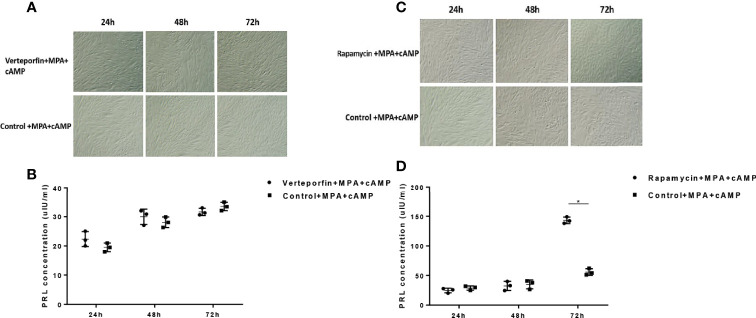
Effects of Verteporfin and Rapamycin treatments on the *in vitro* decidualization of ESCs. Compared to the cultured control group, the morphology of ESCs in the Verteporfin treatment group had no obvious decidual changes after 24 h, 48 h, and 72 h of *in vitro* induction **(A)**. ELISA results suggested that there was no significant difference in the dPRL levels in the culture medium of ESCs in the groups of Verteporfin and the control group after 24 h, 48 h, and 72 h of decidualization (*p* > 0.05) **(B)**. After 24 h and 48 h of *in vitro* induction, some of the ESCs in the Rapamycin-treated and control groups were rounded and enlarged. Seventy-two hours after *in vitro* induction, compared with the control group, the Rapamycin-treated ESCs showed obvious decidual-like changes **(C)**. After 72 h of decidualization, the dPRL level was significantly increased in the cell culture medium of the Rapamycin group compared with that of the control group (*p* < 0.05) **(D)**. *p < 0.05.

To explore the effect of the YAP-autophagy signal on the *in vitro* decidualization of ESCs, we further induced decidualization by Verteporfin and Rapamycin treatments or negative controls (DMSO) exposed to decidual induction *in vitro*. The results showed that compared to the cultured control group, the morphology of most ESCs in the Verteporfin treatment group had no obvious changes after 24 h, 48 h, and 72 h of *in vitro* induction, and a large number of ESCs showed a spindle-like shape. After only 72 h of induction, a small number of ESCs in the control group were rounded and enlarged, with slight decidual-like changes, whereas the ESCs of the Verteporfin group did not change significantly (**Figure 6A**). ELISA results suggested that there was no significant difference in the dPRL levels of decidualization marker in the culture medium of ESCs in the groups of Verteporfin and the control group after 24 h, 48 h, and 72 h of decidualization (*p* > 0.05) (**Figure 6B**).

Regarding the Rapamycin group, compared with the control group, most ESCs in the Rapamycin-treated group were spindle shaped 24 h after *in vitro* decidualization. After 48 h of *in vitro* induction, some of the ESCs in the Rapamycin-treated and control groups were rounded and enlarged. Seventy-two hours after *in vitro* induction, compared with the control group, the Rapamycin-treated ESCs showed obvious decidual-like changes, with a large number of cells becoming round, enlarged, and rich in cytoplasm (**Figure 6C**). ELISA results suggested that there was no significant difference in the dPRL level of decidualized marker between the Rapamycin group and control group after 24 h and 48 h of decidualization (*p* > 0.05). After 72 h of decidualization, the dPRL level was significantly increased in the cell culture medium of the Rapamycin group compared with that of the control group (*p* < 0.05) (**Figure 6D**).

## Discussion

In this study, we first found a significant correlation in the YAP interaction set of 155 genes through bioinformatics analysis, from the GSE51981 chip samples of endometriosis-associated research (Endometriosis vs. Non-Endometriosis), analyzed the GO enrichment analysis and KEGG pathway enrichment, and found that these genes interact with YAP and the enrichment of autophagy function significantly; YAP may be correlated with cell autophagy in EMS.

On this basis, *in vitro* cell experiments showed that the protein level of the negative regulator of autophagy, mTOR, was significantly increased in the eutopic endometrial stromal cells in EMS than in the normal endometrial stromal cells, while the ratio of autophagy marker protein LC3-II/LC3-I was significantly decreased in the eutopic ESCs than in the normal endometrial stromal cells. When autophagy is forming, cytoplasmic LC3 (LC3-I) will enzymatically degrade a small polypeptide and transform into membrane-type autophagosome (LC3-II), which means that LC3-II will increase significantly in autophagy cells (31). Therefore, the high expression of negative autophagy regulatory factor mTOR and the reduced LC3-II/LC3-I ratio in the eutopic endometrial stromal cells indicated that the autophagy level of the endometrial stromal cells in EMS was significantly inhibited. To the extent of protein locations, our immunofluorescence showed the protein locations of YAP and mTOR in the eutopic ESCs from the nucleus to cytoplasm and *vice versa*. It suggests that YAP is activated after nuclear localization and is combined with related transcription factors. At this time, the Hippo-YAP signaling pathway is activated to exert its regulatory role on downstream genes. Similarly, the increased expression of mTOR in the cytoplasm of eutopic ESCs indicates that the mTOR pathway was active, and the autophagy level was impaired in the eutopic ESCs. This is consistent with the results of studies on the decreased level of autophagy in the eutopic and ectopic endometrium tissues in many cases of EMS (20, 32). Based on these findings, we speculated that there is a deeper connection between the Hippo-YAP signaling pathway and autophagy in EMS, and mTOR may be a key mediator molecule.

Many oncology studies have shown that YAP is involved in the regulation of autophagy signals (33–35). For example, YAP reduces cisplatin-induced apoptosis by activating autophagy in ovarian cancer cells (33). In undifferentiated pleomorphic sarcoma cells, YAP inhibits autophagy independently of NF-κB signaling (35). Studies on gastric cancer have found that the downregulation of YAP truncates weak mitochondrial autophagy, leading to the apoptosis of gastric cancer cells (36). In addition, YAP also promotes multi-drug resistance of liver cancer cells through the RAC1-Ros-MTOR pathway, thereby inhibiting autophagy-related cell death (26). So, does the increased expression of YAP in endometrial stromal cells in EMS participate in the regulation of the weakened autophagy signal in endometrial cells? In the following studies, we intend to conduct in-depth studies on the regulation mechanism of YAP on autophagy in the eutopic endometrial stromal cells in EMS and the biological functions involved in both.

To further clarify the regulatory relationship between YAP signal and autophagy in EMS, a YAP overexpression experiment was conducted in endometrial stromal cells in EMS. mTOR and autophagy levels were detected by direct interference with YAP expression. The results showed that the mTOR protein level and lc3-II/LC3-I ratio in the eutopic ESCs increased after the overexpression of YAP. Since autophagosomes are subcellular structures, the formation of autophagosomes cannot be observed under an ordinary light microscope, so direct observation of autophagosomes requires TEM. As the gold standard for the detection of autophagy, TEM results showed that the number of autophagosomes in the ESCs overexpressing YAP was significantly reduced compared with the control group, suggesting that the level of autophagy was inhibited. In recent years, several studies have found that YAP and autophagy are closely related. For example, the YAP/TAZ-autophagy axis can control the survival and proliferation of cells (37). As an upstream transcriptional regulator, YAP activates the mTOR pathway and inhibits autophagy (38). Our study revealed the correlation between YAP and autophagy in EMS and opened new ideas and directions for future basic research on signaling pathways in EMS and autophagy function. Combined with our previous finding that the mTOR protein level decreased and the LC3-II/LC3-I ratio significantly increased after silencing YAP in the eutopic ESCs, the level of autophagy was enhanced. In conclusion, this part of the study shows that YAP may be involved in the regulation process of the decreased autophagy level of the eutopic ESCs in EMS through the upregulation of the mTOR pathway, thus participating in the occurrence and development of EMS.

YAP is the main effector molecule of the Hippo signaling pathway. The Hippo signaling pathway has extensive and complex cross-linking with many other signaling pathways, and its different effects depend on different cells, different external environments, different co-activators, and different feedback of various upstream and downstream factors. Since YAP has no DNA binding region, it needs to be combined with TEADs or other transcription factors to participate in the regulation of downstream gene expression (39). Studies have found (40) that TEAD binding sites on prolactin (PRL) promoters play an important role in maintaining the basal level of PRL promoter activity, and the overexpression of TEAD1 inhibits the expression of PRL in human decidual cells, which may be realized through the interaction with other transcription factors. In 2017, our group found that YAP was highly expressed in decidual cells and promoted decidualization of ESCs cultured *in vitro*, indicating that YAP is involved in the regulation of normal endometrial stromal cell decidualization (41). A study published in *Hum Reprod* (42) found that decidual induction was performed in immortalized human endometrial stromal cells, and the level of autophagy marker protein LC3-II was significantly increased in decidual cells compared with the control group, indicating that the decidual process of endometrial stromal cells may be related to cell autophagy. Therefore, we speculated that the YAP-autophagy signal may be involved in the regulation of decidualization of the eutopic ESCs in endometriosis and play an important role in embryo implantation.

Previous studies have confirmed that EMS has decreased receptivity in the endometrium, and numerous molecules and signal pathways are involved in the regulation of decidualization of ESCs (11, 43). Local inflammation, stromal differentiation, and improper endometrial reconstruction of endometrium in endometriosis all lead to the endometrial condition that embryo implantation is not acceptable (8, 12). In this study, it has been found that the decidualization of the eutopic ESCs after treatment by Verteporfin, a YAP signal inhibitor, has not affected the decidualization of eutopic ESCs. The decidualization of the endometrium is a very complex and delicate process, which is regulated by many factors, including cytokines, immune cells, and hormones, and accompanied by various epigenetic changes during decidualization. In addition, the molecular and biological characteristics of the endometrium in EMS are fundamentally different from those in non-endometriosis patients (44–46). Therefore, it is understandable that Verteporfin does not change the decidual process of ESCs in endometriosis. As a non-photosensitizer, it inhibits the transcription and translation of YAP and destroys the complex formed by YAP and other downstream transcription factors. However, this blocking effect does not affect or participate in the decidual regulation process of endometrial stromal cells. Based on the former part of the negative regulation effect of YAP-mTOR signals on cell autophagy in the eutopic ESCs, we treated the eutopic ESCs with mTOR inhibitor Rapamycin (autophagy inducer), and induced decidual cells *in vitro*. It is found that Rapamycin had no significant effect on decidualization of ESCs after 24-h and 48-h induction, but Rapamycin treatment promoted the decidualization of the eutopic ESCs after 72 h, and this difference may be due to the induction of decidual function caused by delay and cell gradually. At present, there is no unanimous conclusion on the effect of mTOR autophagy on endometrial decidualization. Studies have reported (47–49) that the mTOR pathway plays an important role in the early embryo implantation process, and the activation of the mTOR pathway promotes NM23 to regulate the decidualization process of mouse and human endometrium. It was also found (42) that the autophagy level was upregulated during decidualization of the endometrium in mice, which was consistent with our findings. In other words, after the administration of the autophagy-inducing agent Rapamycin, decidual changes in the eutopic ESCs were significant, which may be related to the regulation of Rapamycin on the autophagy of the eutopic ESCs.

This study preliminarily explored the effect of the YAP-autophagy signal on the decidualization of the eutopic ESCs and found that the decidual process of the eutopic ESCs was promoted after inhibiting mTOR activity and activating autophagy, suggesting that autophagy may be involved in the regulation of decidualization of the eutopic ESCs in endometriosis. However, whether the increased YAP in the eutopic ESCs of patients with EMS-associated infertility is directly involved in the regulation of decidualization of the endometrium, how enhanced autophagy level regulates the process of decidualization of the endometrium, and whether there are other pathways involved are still unknown and need to be further studied.

There are still some limitations in this study. For example, the mechanisms of YAP regulation of autophagy in the eutopic ESCs was explored *in vitro* only in patients with EMS-associated infertility, which would be more convincing if validated in an *in vivo* model. In addition, any signaling pathway is not a single one, and there are extensive crossover and network-like interactions among them, and a certain pathway cannot explain all the research problems. In the future, more transcriptomics and proteomics studies are needed to study the histological and cellular structure of the endometrium, as well as the interactions between genes, proteins and molecules from an overall perspective, so as to provide new ideas for exploring the pathogenesis of EMS.

Therefore, subsequent studies will continue to explore the regulatory effects of the YAP-autophagy signal on the decidual and endometrial receptivity of ESCs, to provide a theoretical basis for exploring the pathogenesis of EMS and improving endometrial receptivity and provide new options for the treatment of endometriosis-associated infertility.

## Data Availability Statement

The datasets presented in this study can be found in online repositories. The names of the repository/repositories and accession number(s) can be found in the article/**Supplementary Material**.

## Ethics Statement

This study was approved by the Ethics Committee of West China Second University Hospital of Sichuan University. The patients/participants provided their written informed consent to participate in this study.

## Author Contributions

TP performed bioinformatic analysis and immunofluorescence and wrote the manuscript, which was commented on by all authors. BL performed the cell transfection and *in vitro* decidualization experiments. WH evaluated all data and contributed to discussion. DL, YL, and LX analyzed the qRT-PCR and WB data. XH and YO contributed to human sample collection. HZ supervised the study and reviewed the manuscript. All authors contributed to the article and approved the submitted version.

## Funding

This work was supported by the National Key R&D Program of China (grant number 2017YCF1001200); The National Natural Science Foundation of China (grant number 82101719); The National Natural Science Foundation of China (grant number 82071625); The Key Project of Department of Science and Technology of Sichuan (grant number 2018SZ0124); and the International Cooperation of Chengdu Science and Technology Bureau (grant number 2017GH0200060HZ).

## Conflict of Interest

The authors declare that the research was conducted in the absence of any commercial or financial relationships that could be construed as a potential conflict of interest.

## Publisher’s Note

All claims expressed in this article are solely those of the authors and do not necessarily represent those of their affiliated organizations, or those of the publisher, the editors and the reviewers. Any product that may be evaluated in this article, or claim that may be made by its manufacturer, is not guaranteed or endorsed by the publisher.
