# Supplementary figures and images for: Increased Expression of YAP Inhibited the Autophagy Level by Upregulating mTOR Signal in the Eutopic ESCs of Endometriosis
Source: Front Endocrinol (Lausanne). 2022 Jan 31;13:813165. doi: 10.3389/fendo.2022.813165 (PMC8842667; doi:10.3389/fendo.2022.813165)

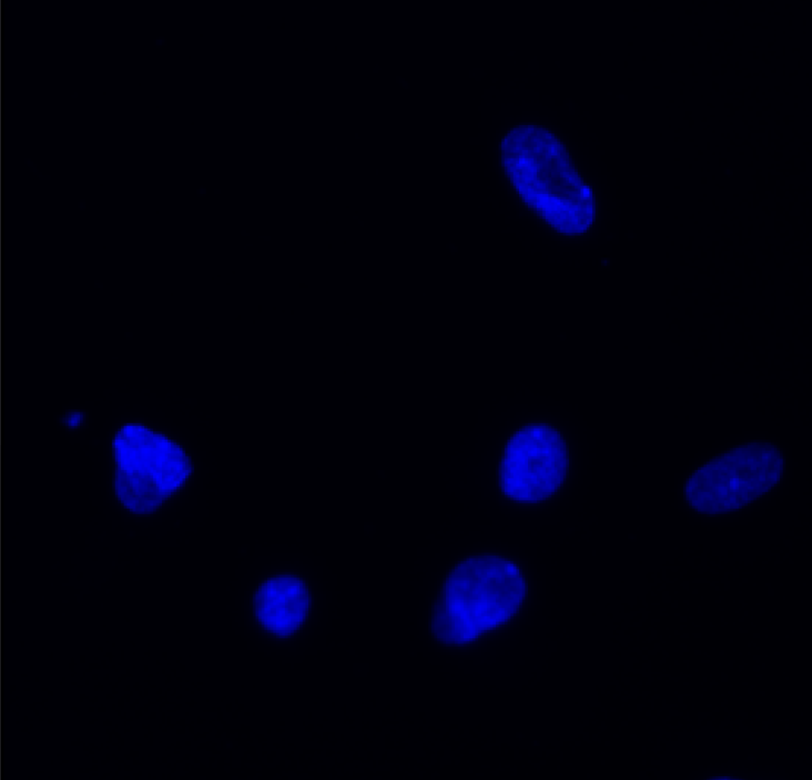

Supplement: Supplementary file 1 [file DataSheet_1.zip › IF mTOR con1.tif]

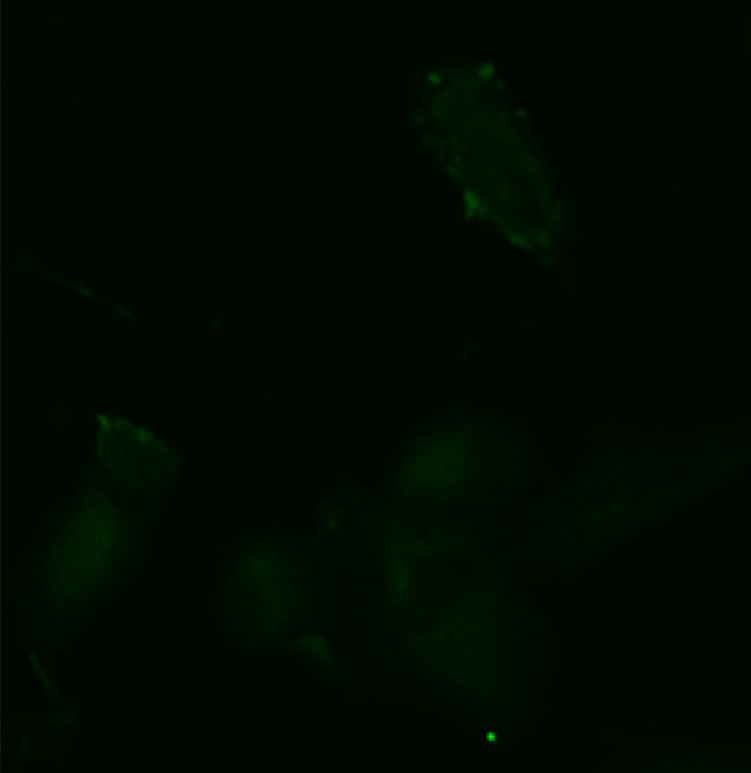

Supplement: Supplementary file 1 [file DataSheet_1.zip › IF mTOR con2.tif]

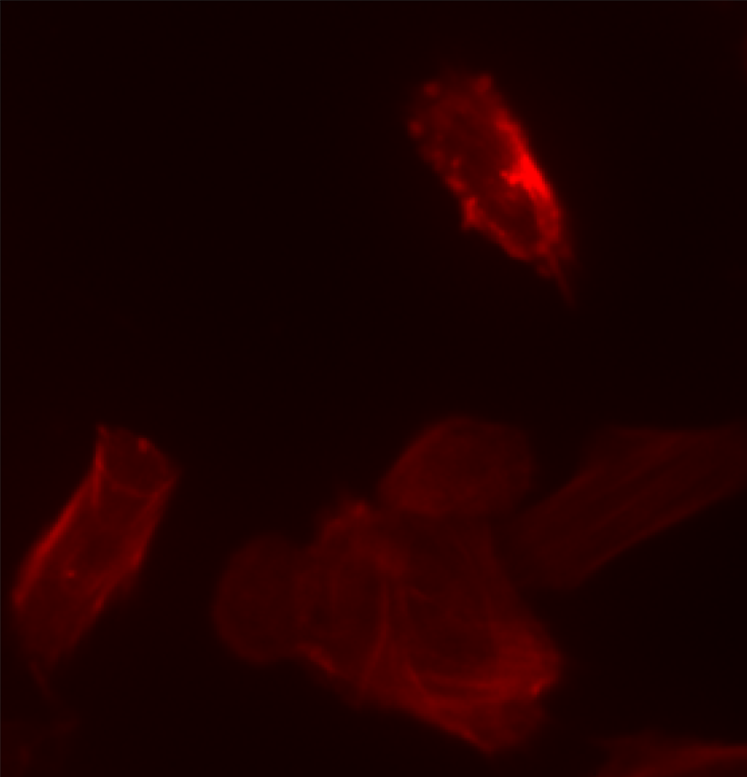

Supplement: Supplementary file 1 [file DataSheet_1.zip › IF mTOR con3.tif]

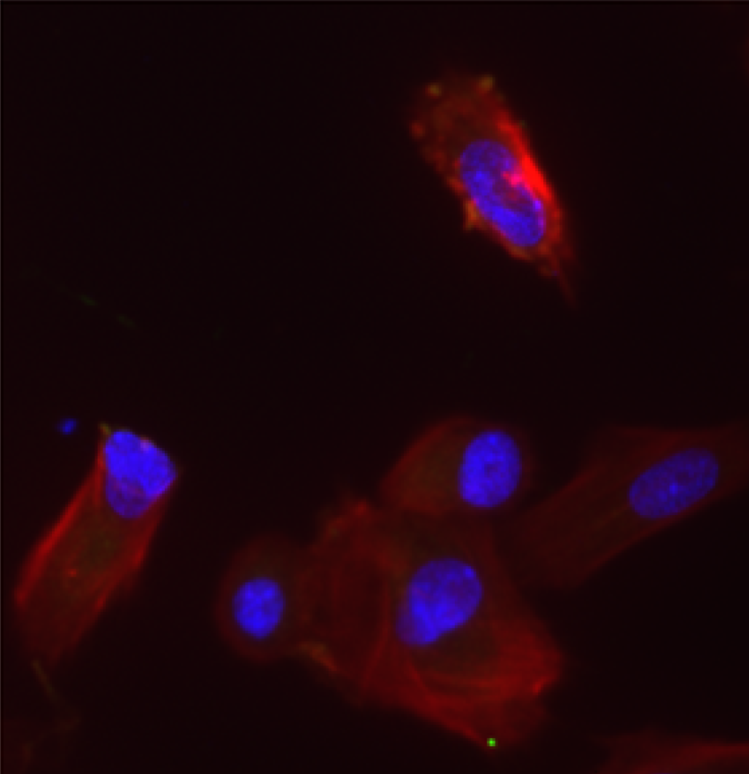

Supplement: Supplementary file 1 [file DataSheet_1.zip › IF mTOR con4.tif]

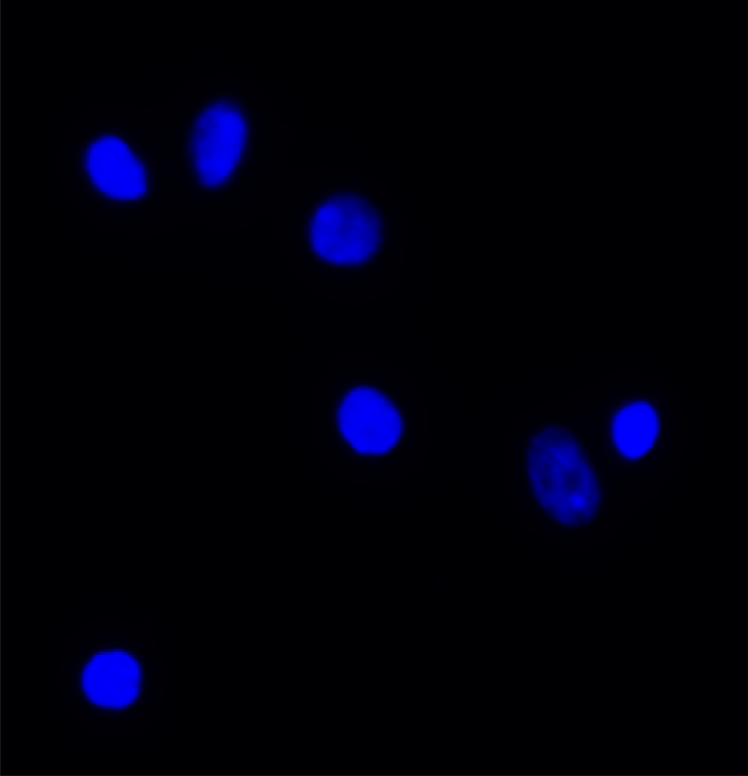

Supplement: Supplementary file 1 [file DataSheet_1.zip › IF mTOR ems1.tif]

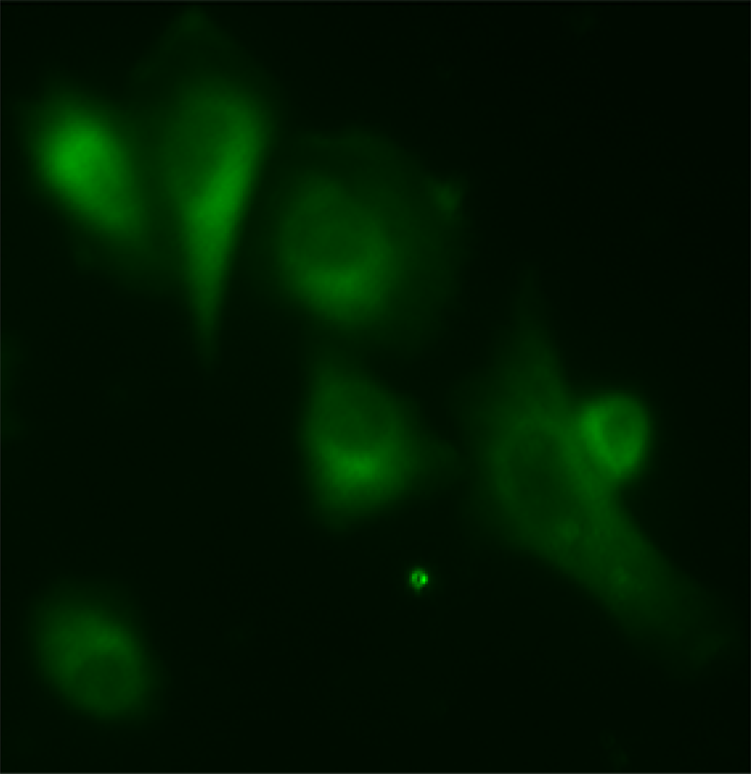

Supplement: Supplementary file 1 [file DataSheet_1.zip › IF mTOR ems2.tif]

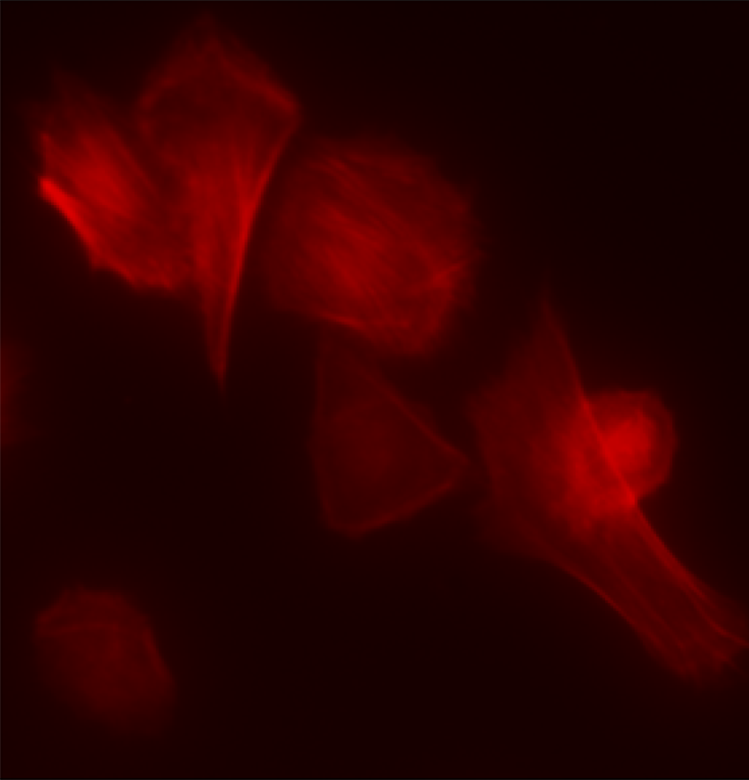

Supplement: Supplementary file 1 [file DataSheet_1.zip › IF mTOR ems3.tif]

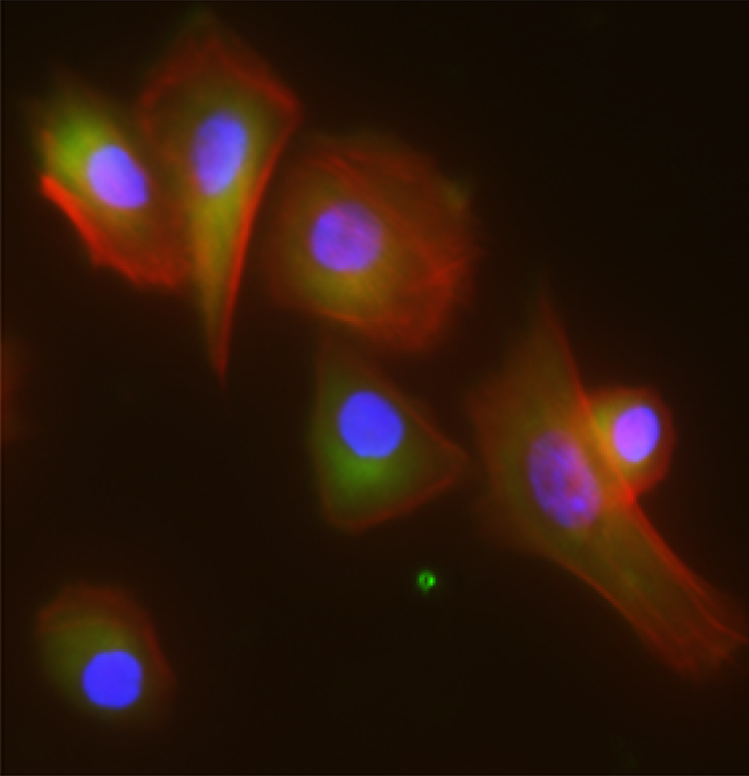

Supplement: Supplementary file 1 [file DataSheet_1.zip › IF mTOR ems4.tif]

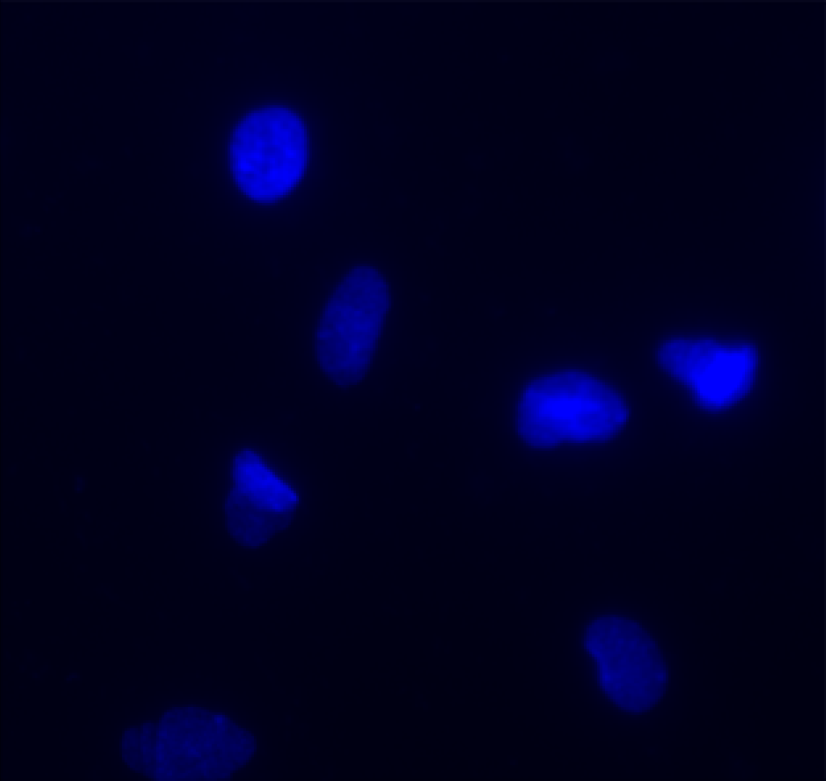

Supplement: Supplementary file 1 [file DataSheet_1.zip › IF YAP con1.tif]

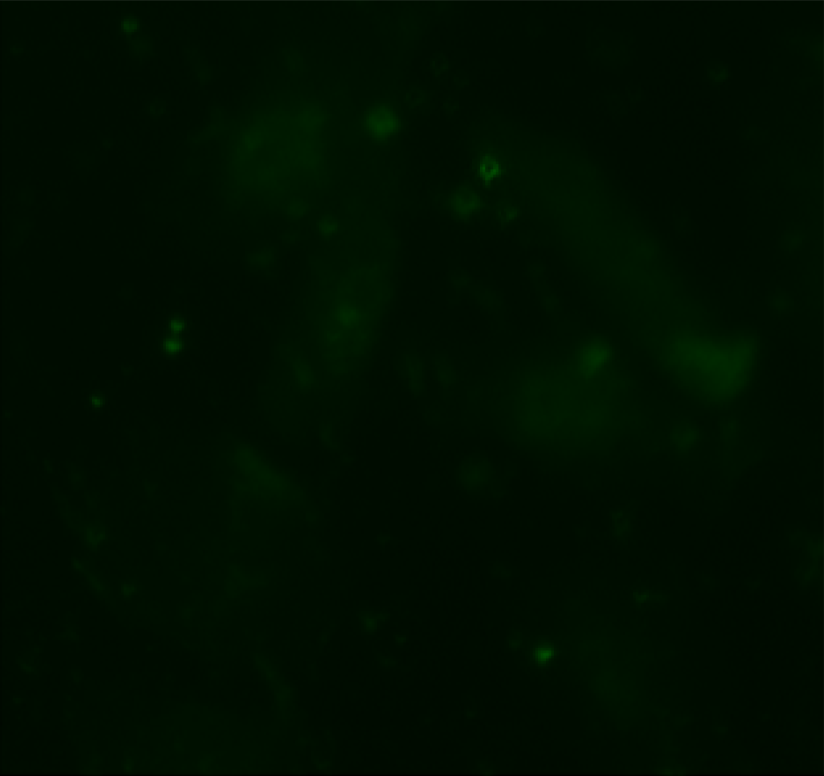

Supplement: Supplementary file 1 [file DataSheet_1.zip › IF YAP con2.tif]

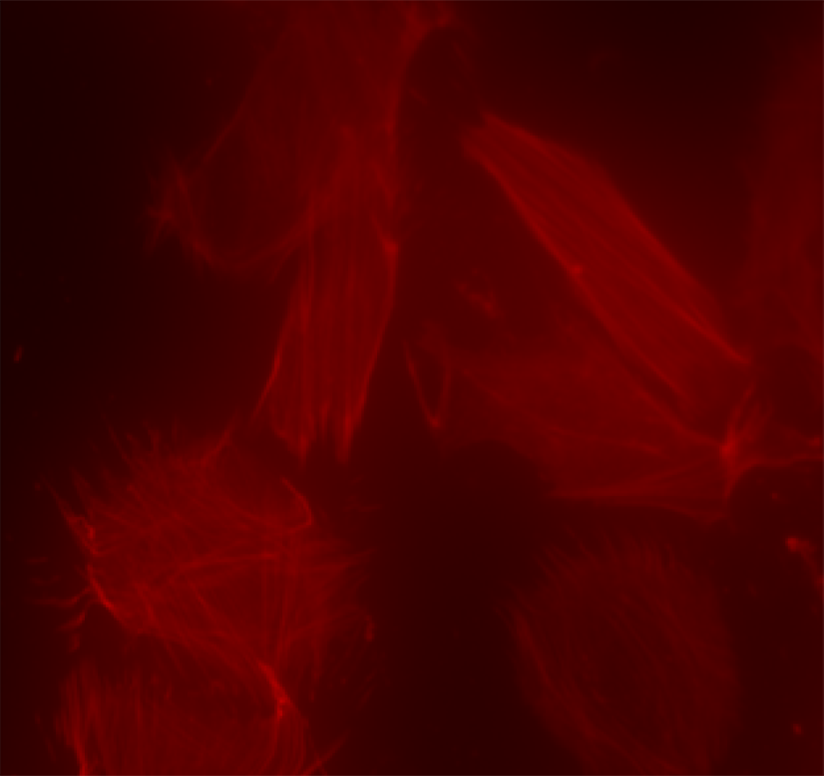

Supplement: Supplementary file 1 [file DataSheet_1.zip › IF YAP con3.tif]

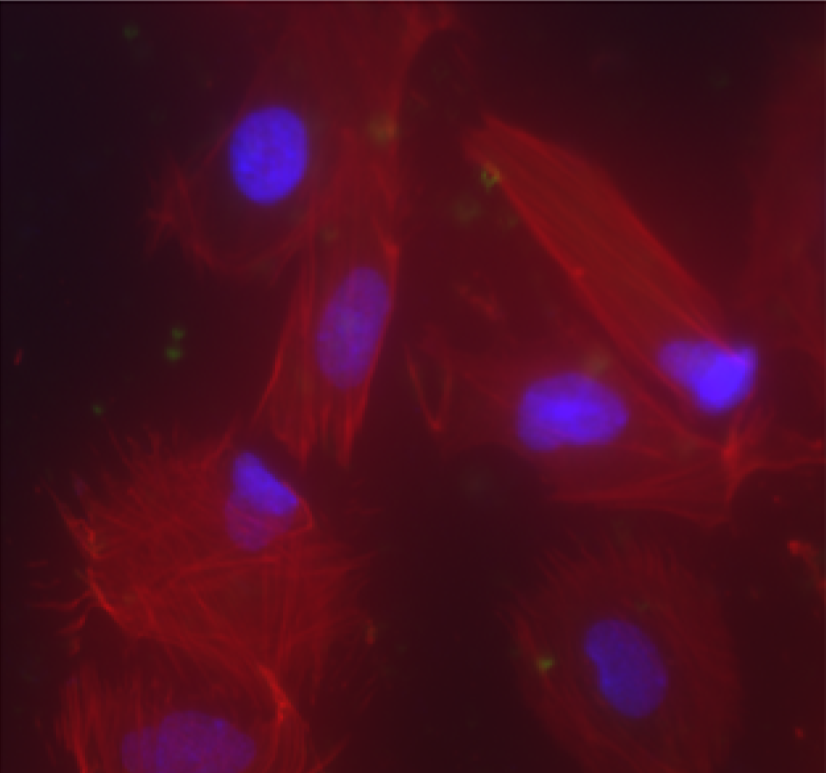

Supplement: Supplementary file 1 [file DataSheet_1.zip › IF YAP con4.tif]

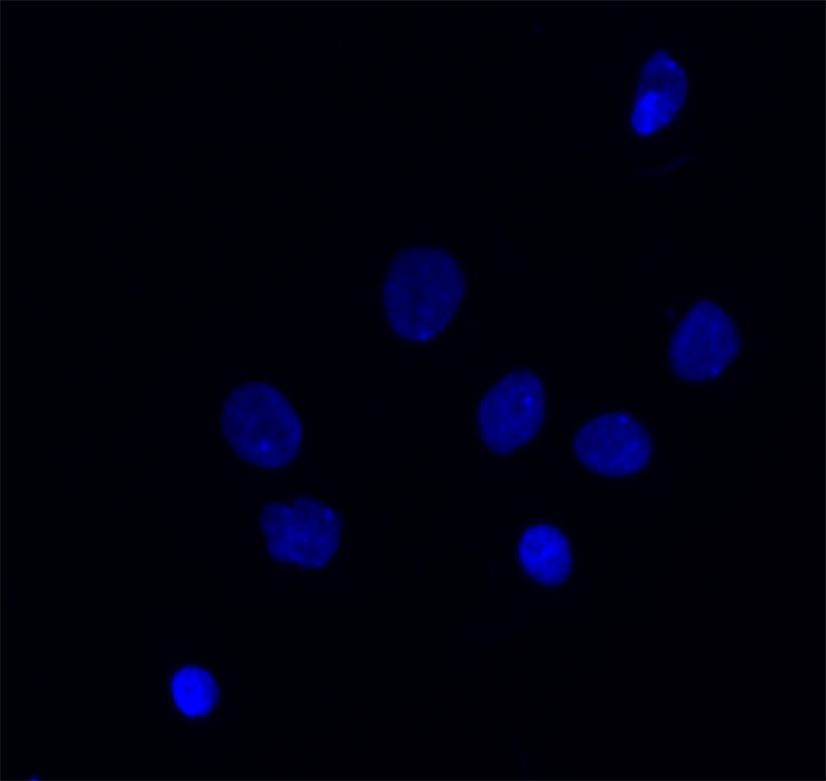

Supplement: Supplementary file 1 [file DataSheet_1.zip › IF YAP ems1.tif]

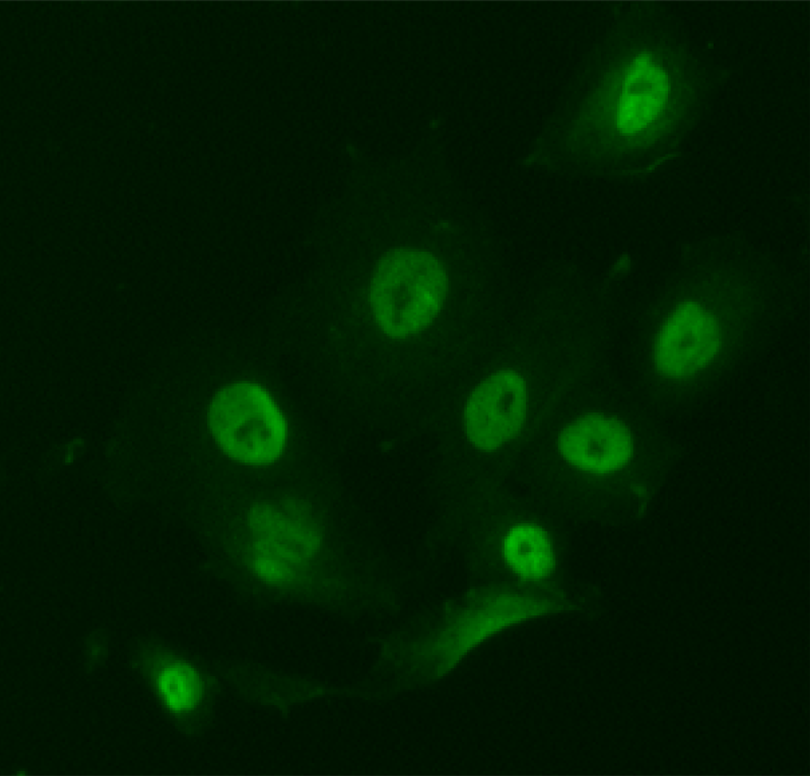

Supplement: Supplementary file 1 [file DataSheet_1.zip › IF YAP ems2.tif]

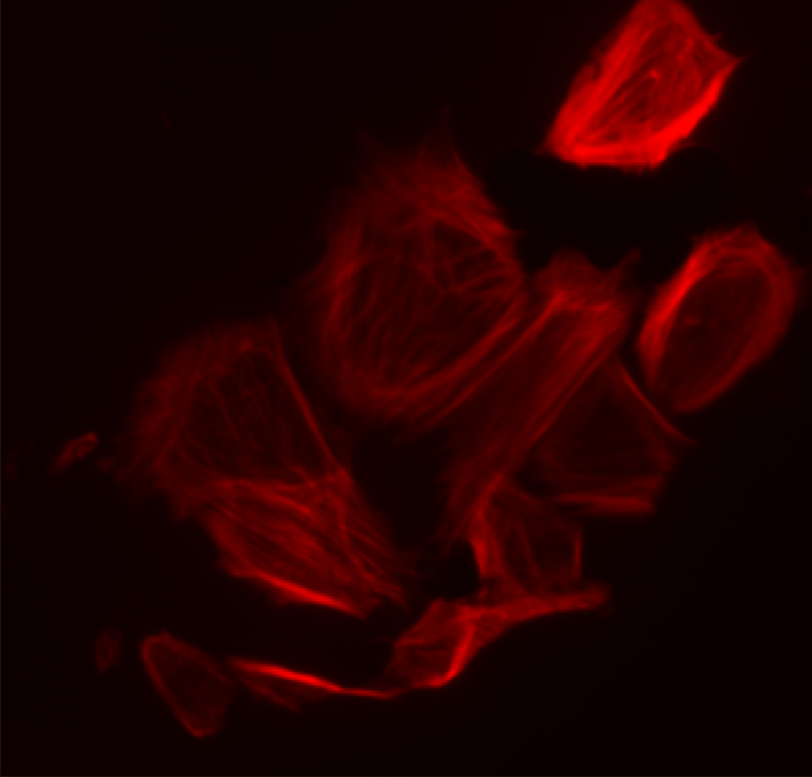

Supplement: Supplementary file 1 [file DataSheet_1.zip › IF YAP ems3.tif]

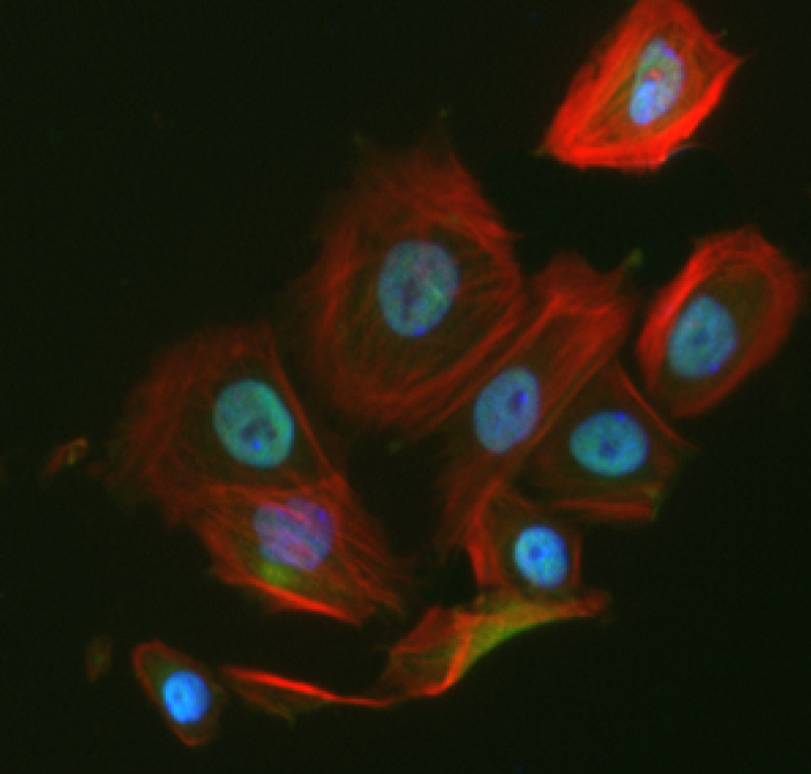

Supplement: Supplementary file 1 [file DataSheet_1.zip › IF YAP ems4.tif]

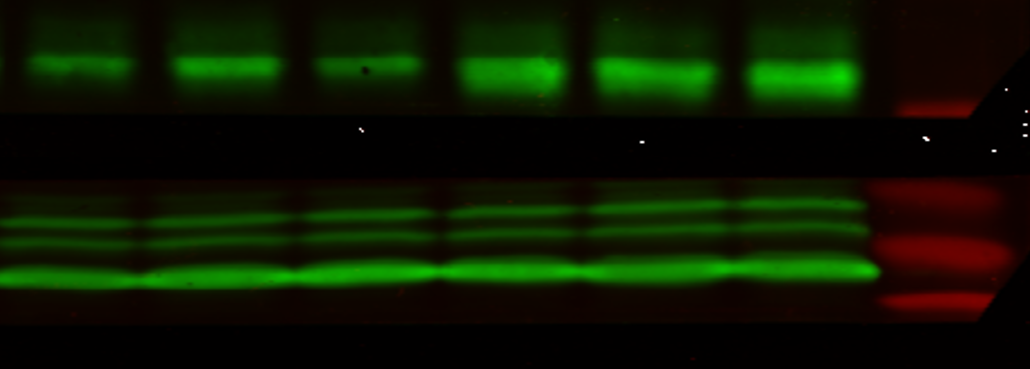

Supplement: Supplementary file 2 [file DataSheet_2.zip › YAP OE-WB/YAP OE1.tif]

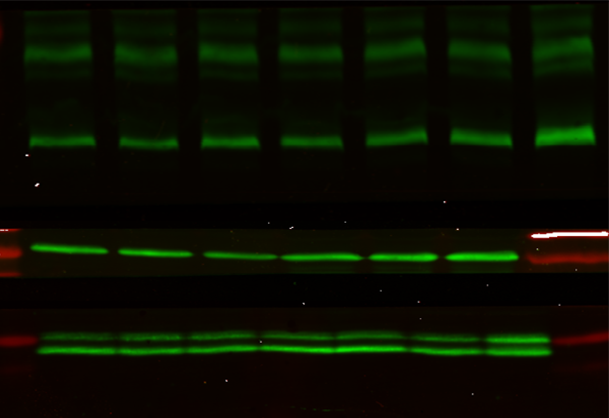

Supplement: Supplementary file 2 [file DataSheet_2.zip › YAP OE-WB/YAP OE2.tif]

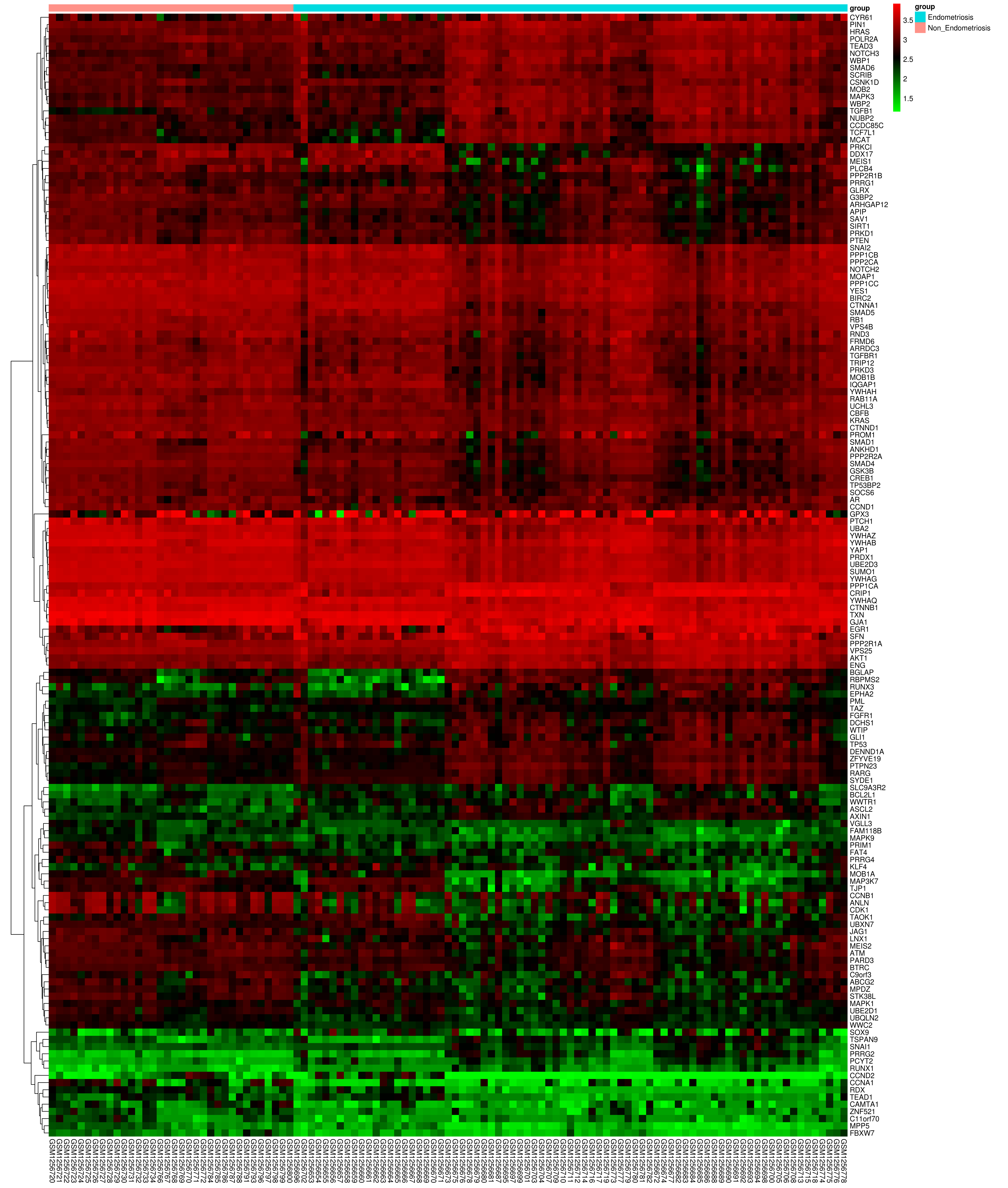

Supplement: Supplementary file 3 [file Image_1.png]

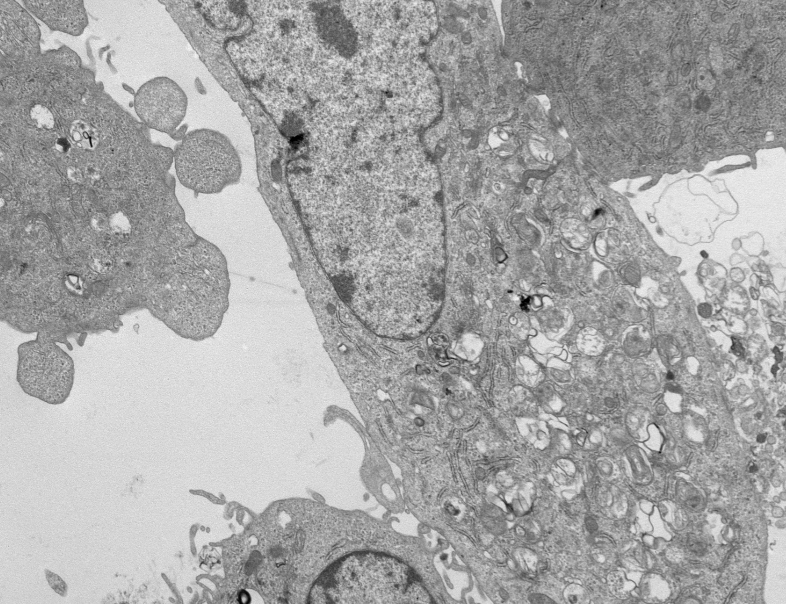

Supplement: Supplementary file 4 [file Image_2.png]

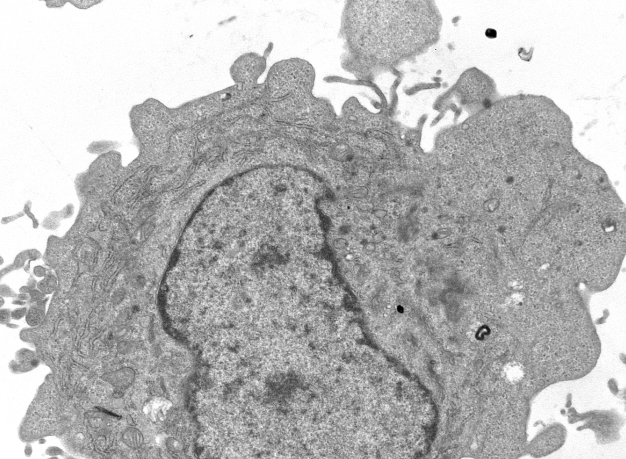

Supplement: Supplementary file 5 [file Image_3.png]

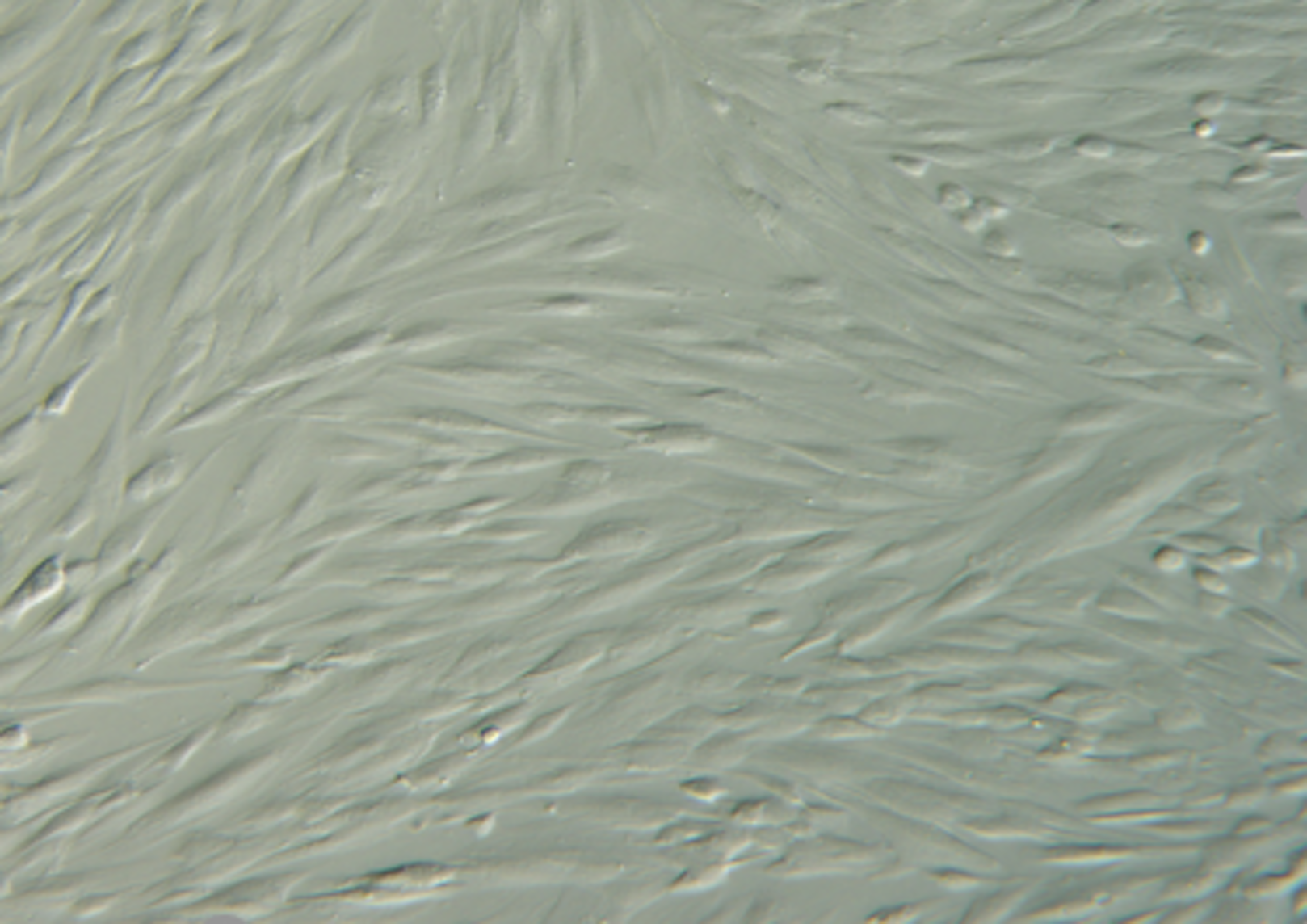

Supplement: Supplementary file 6 [file Image_4.png]

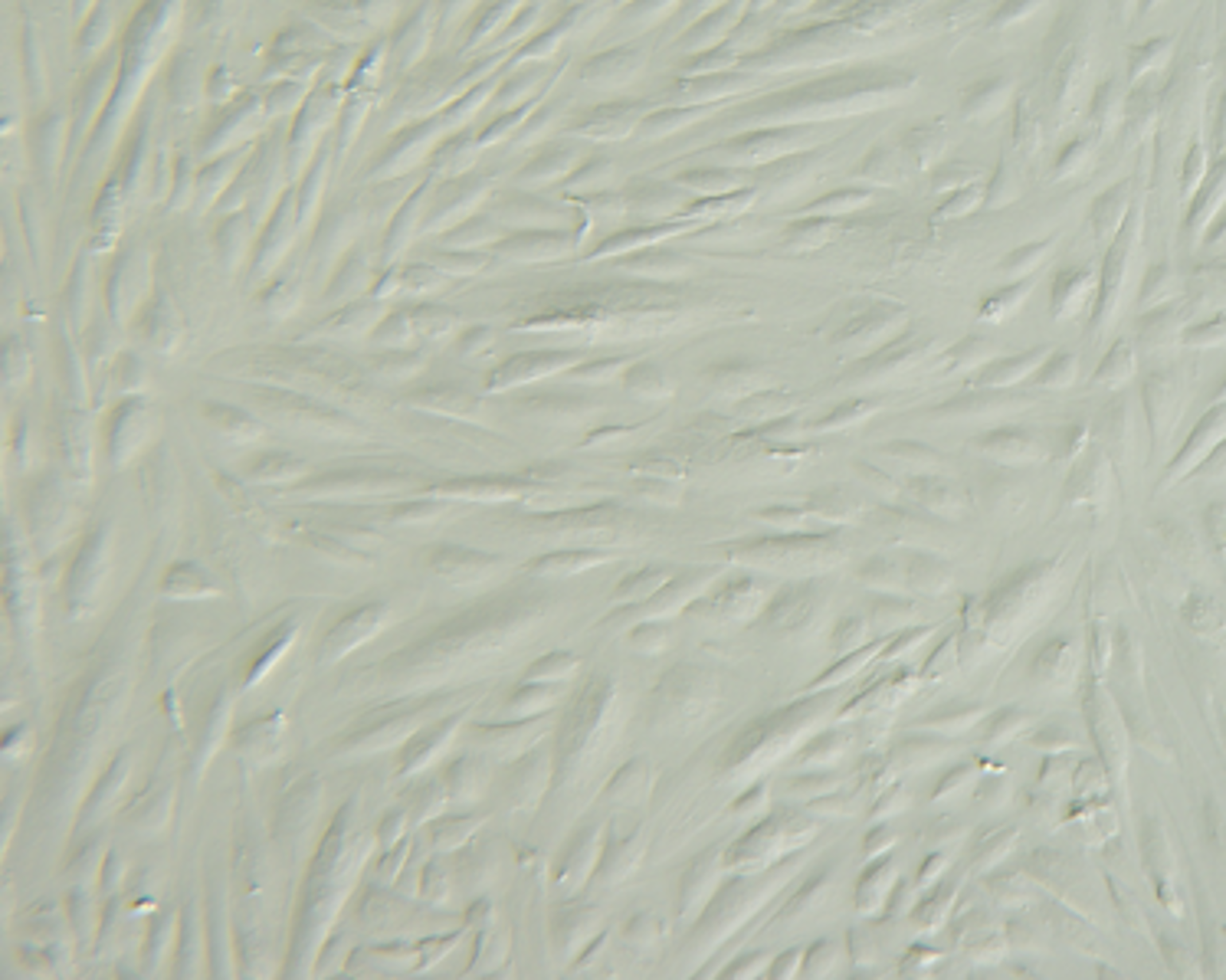

Supplement: Supplementary file 7 [file Image_5.png]

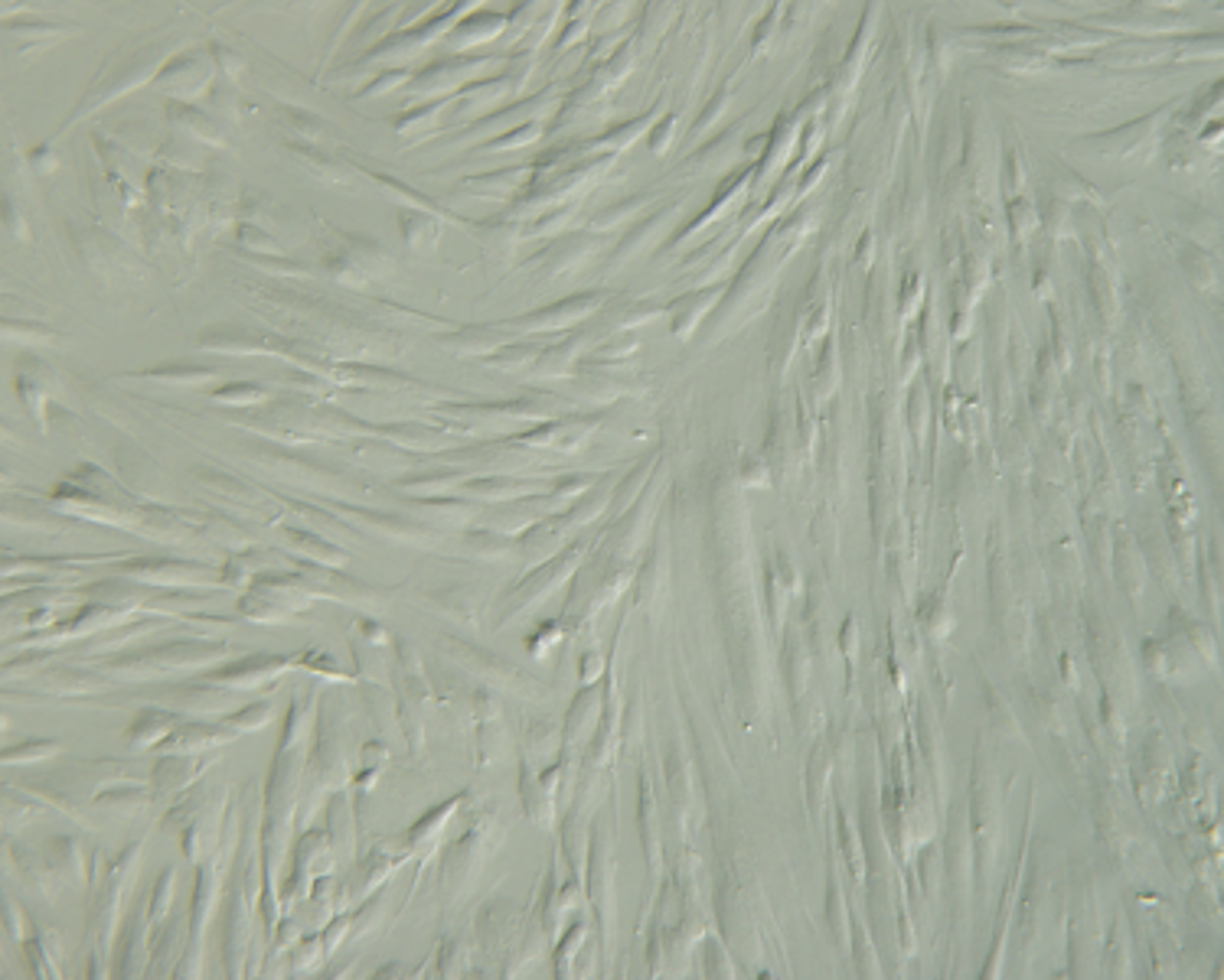

Supplement: Supplementary file 8 [file Image_6.png]

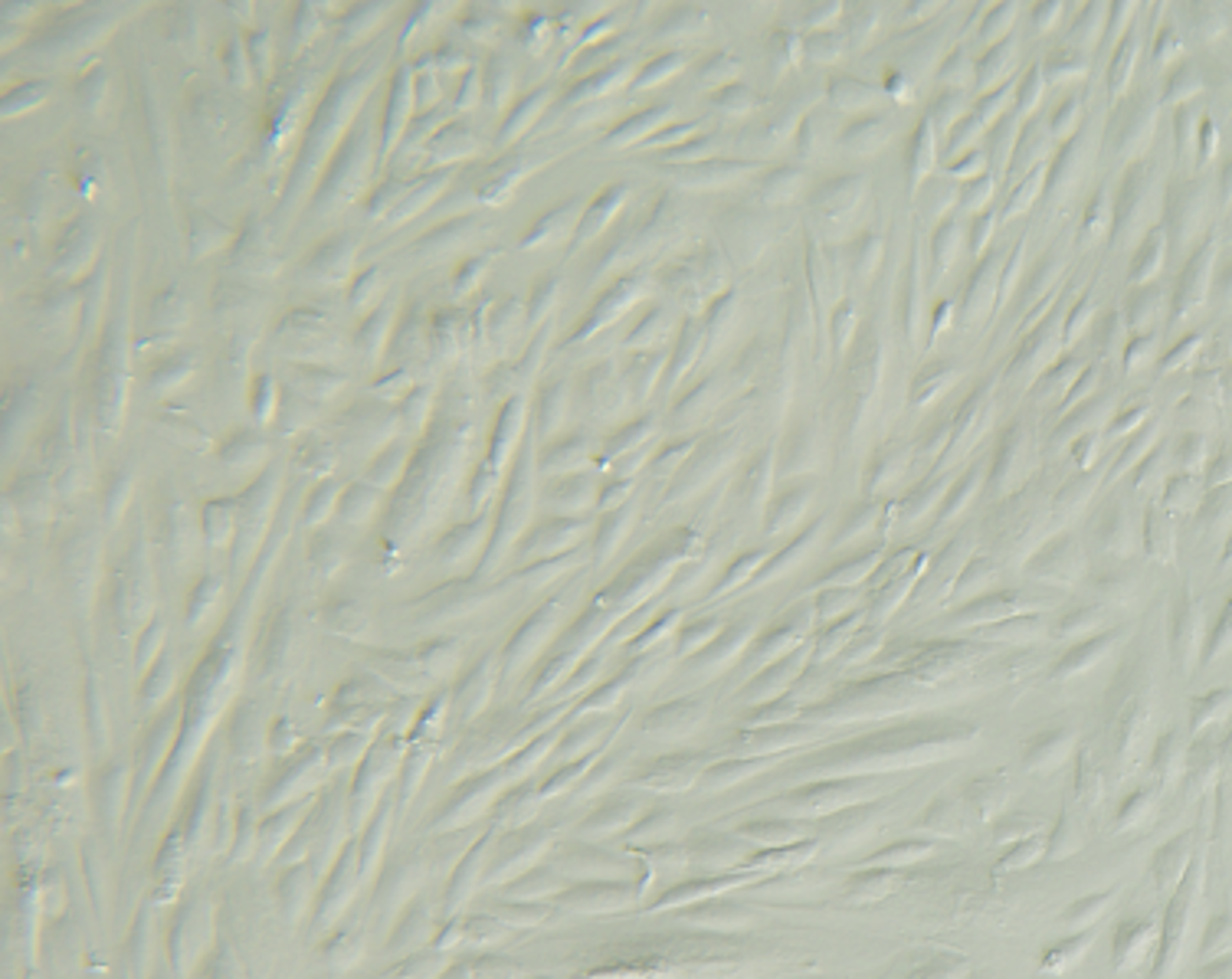

Supplement: Supplementary file 9 [file Image_7.png]

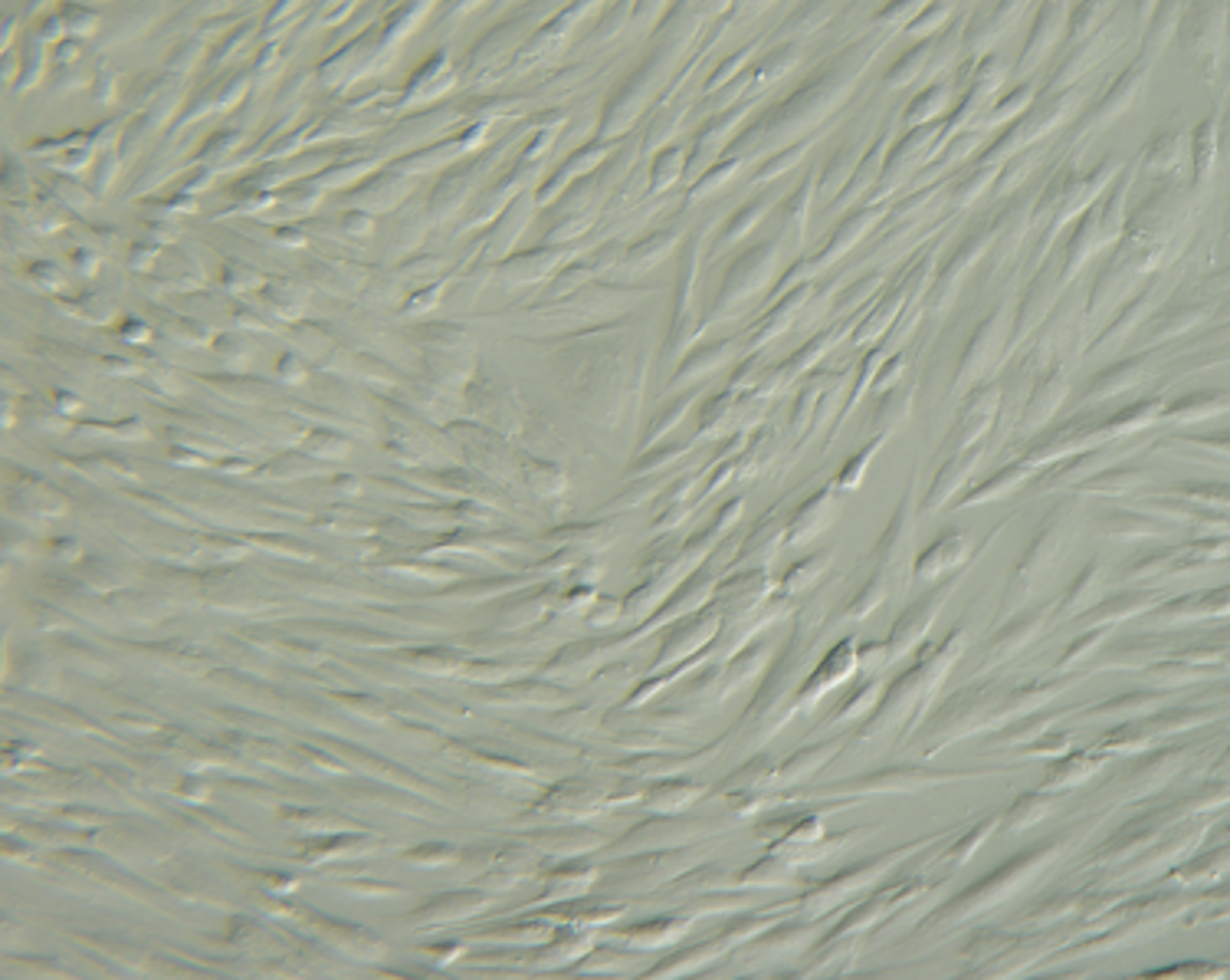

Supplement: Supplementary file 10 [file Image_8.png]

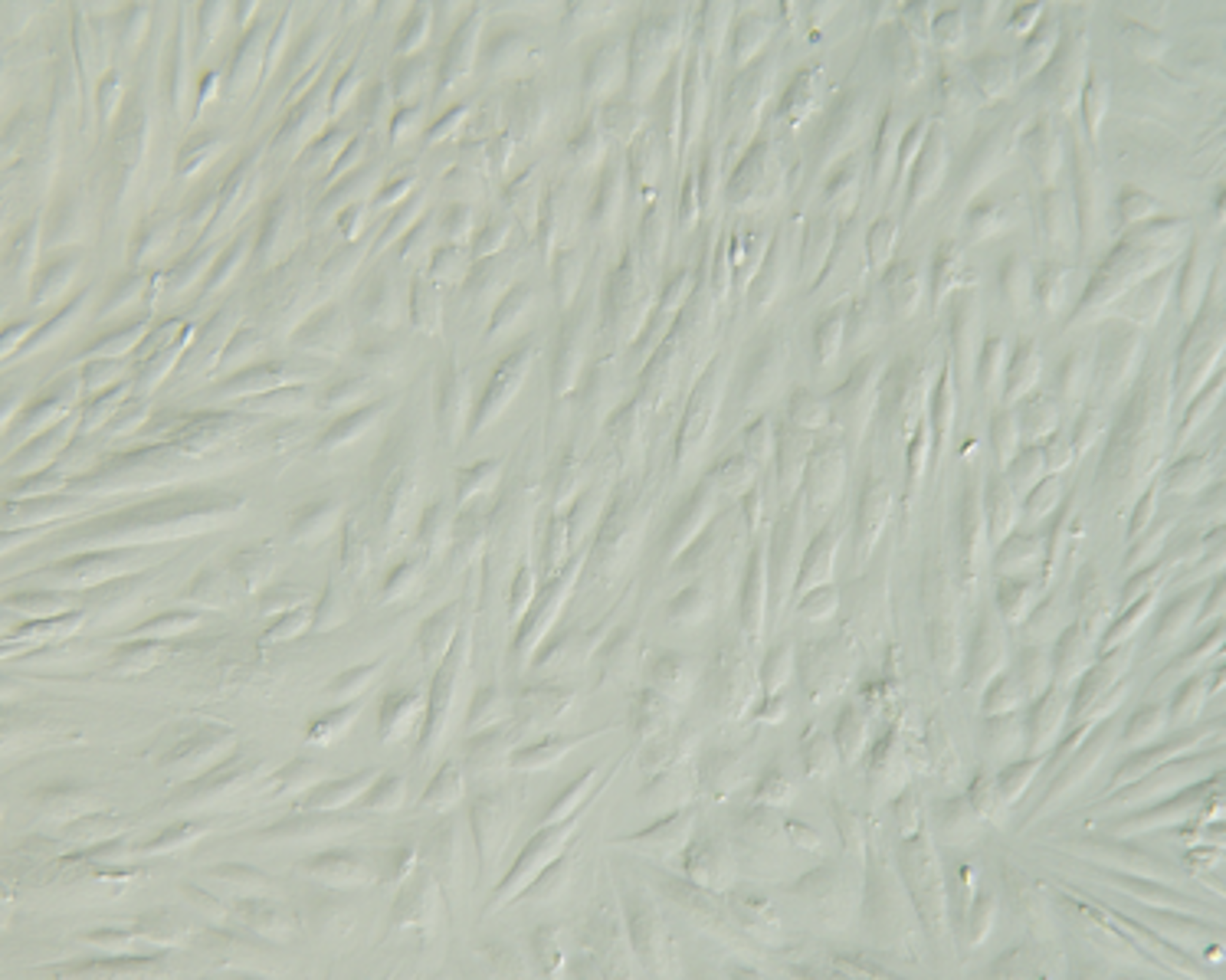

Supplement: Supplementary file 11 [file Image_9.png]

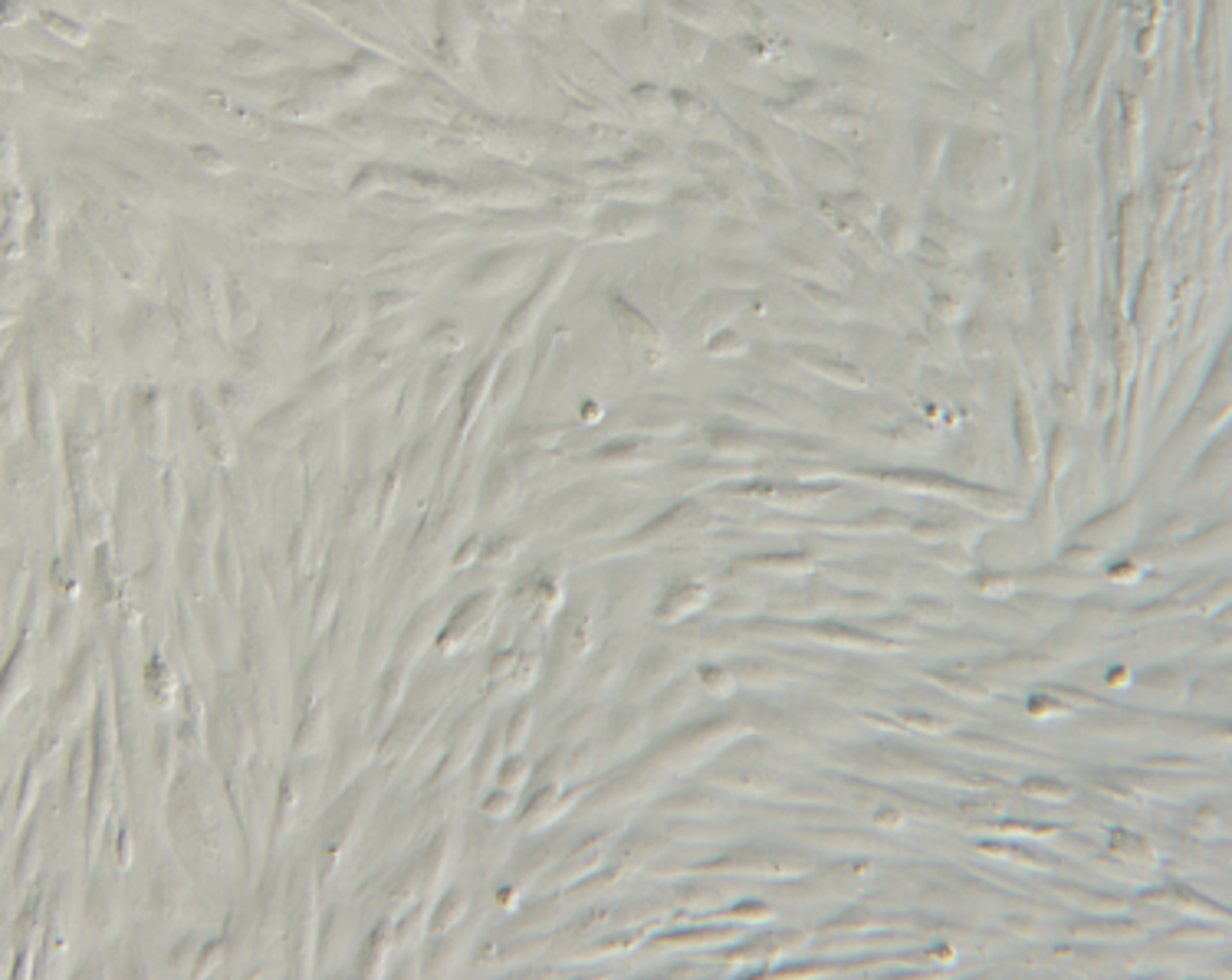

Supplement: Supplementary file 12 [file Image_10.png]

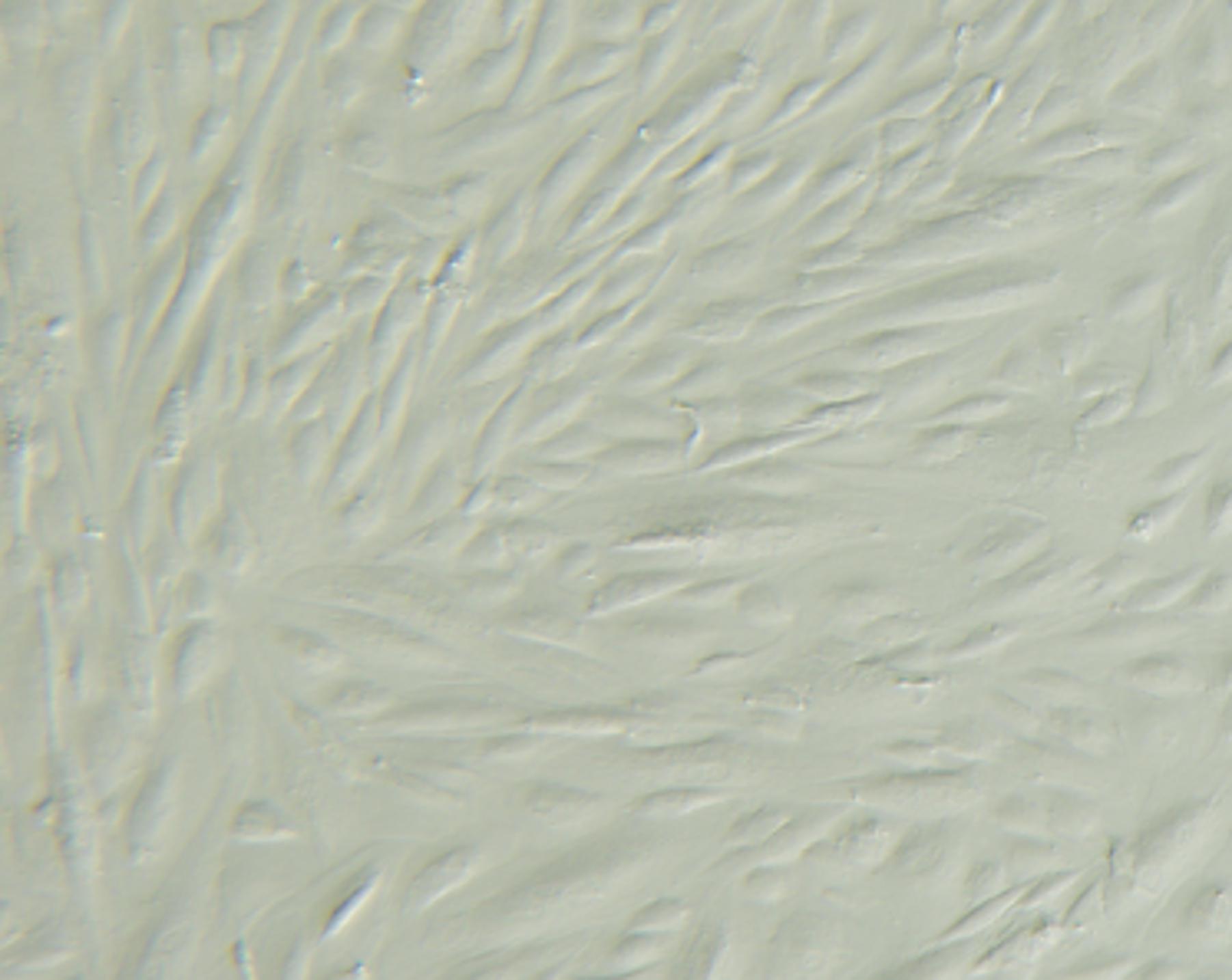

Supplement: Supplementary file 13 [file Image_11.png]

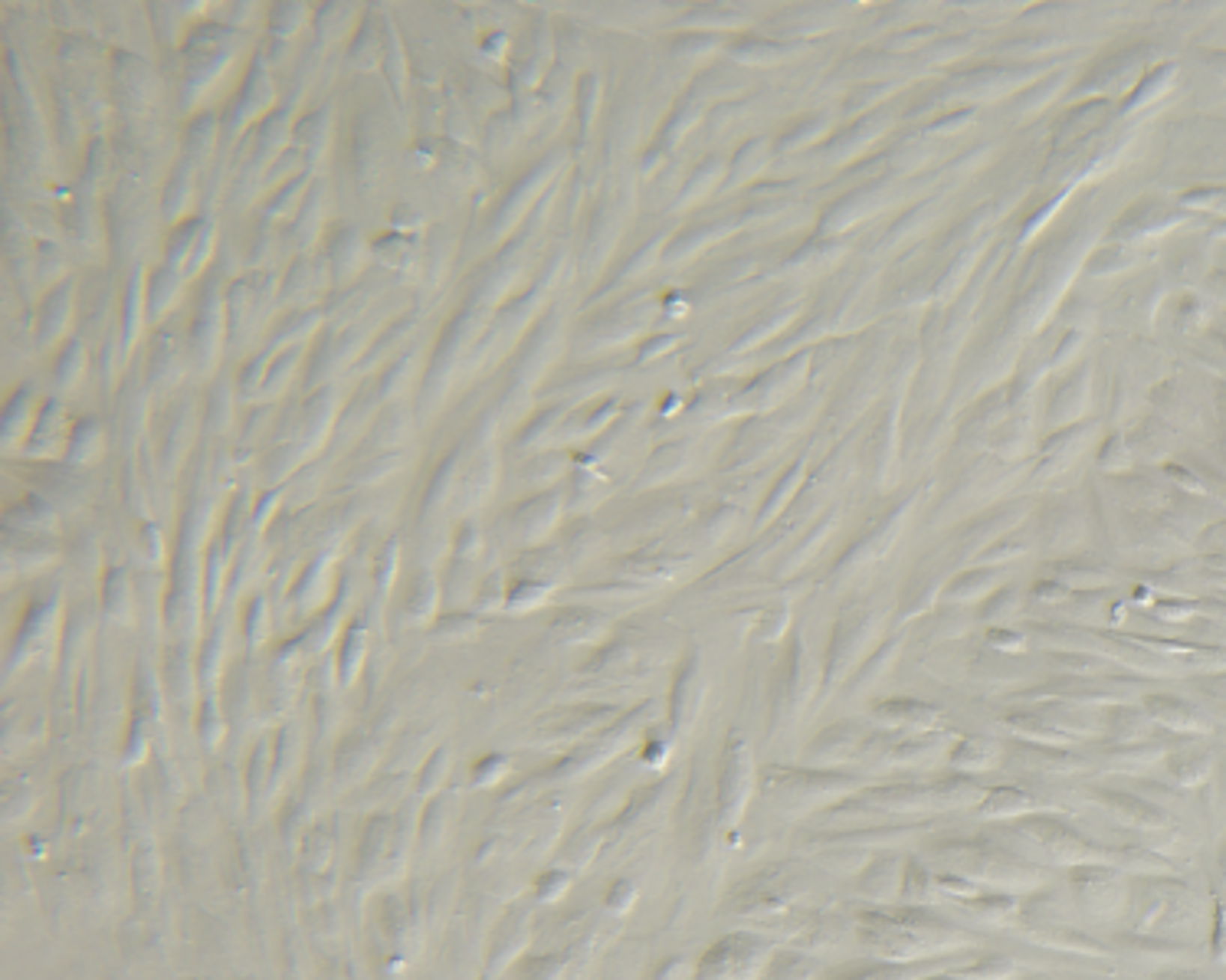

Supplement: Supplementary file 14 [file Image_12.png]

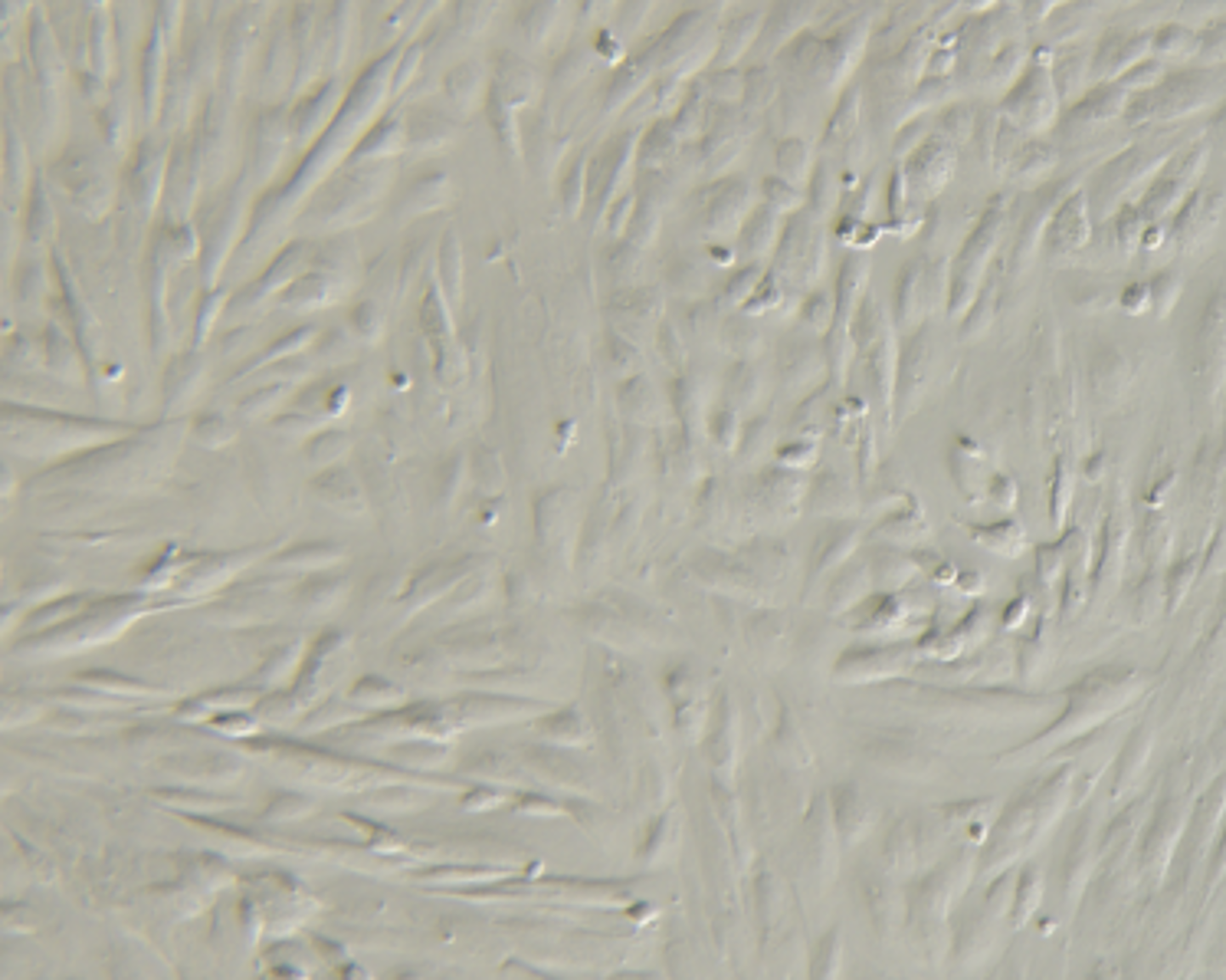

Supplement: Supplementary file 15 [file Image_13.png]

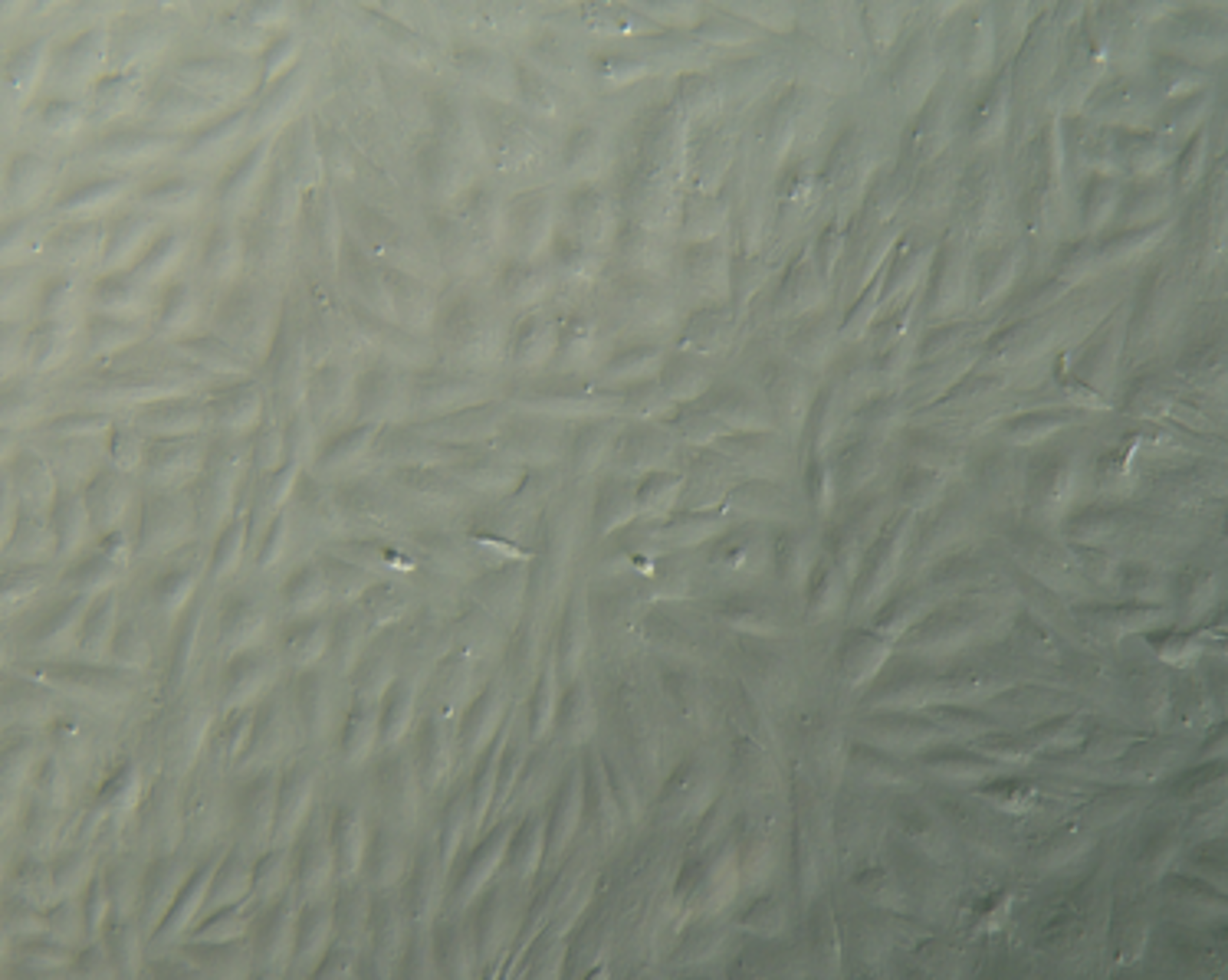

Supplement: Supplementary file 16 [file Image_14.png]

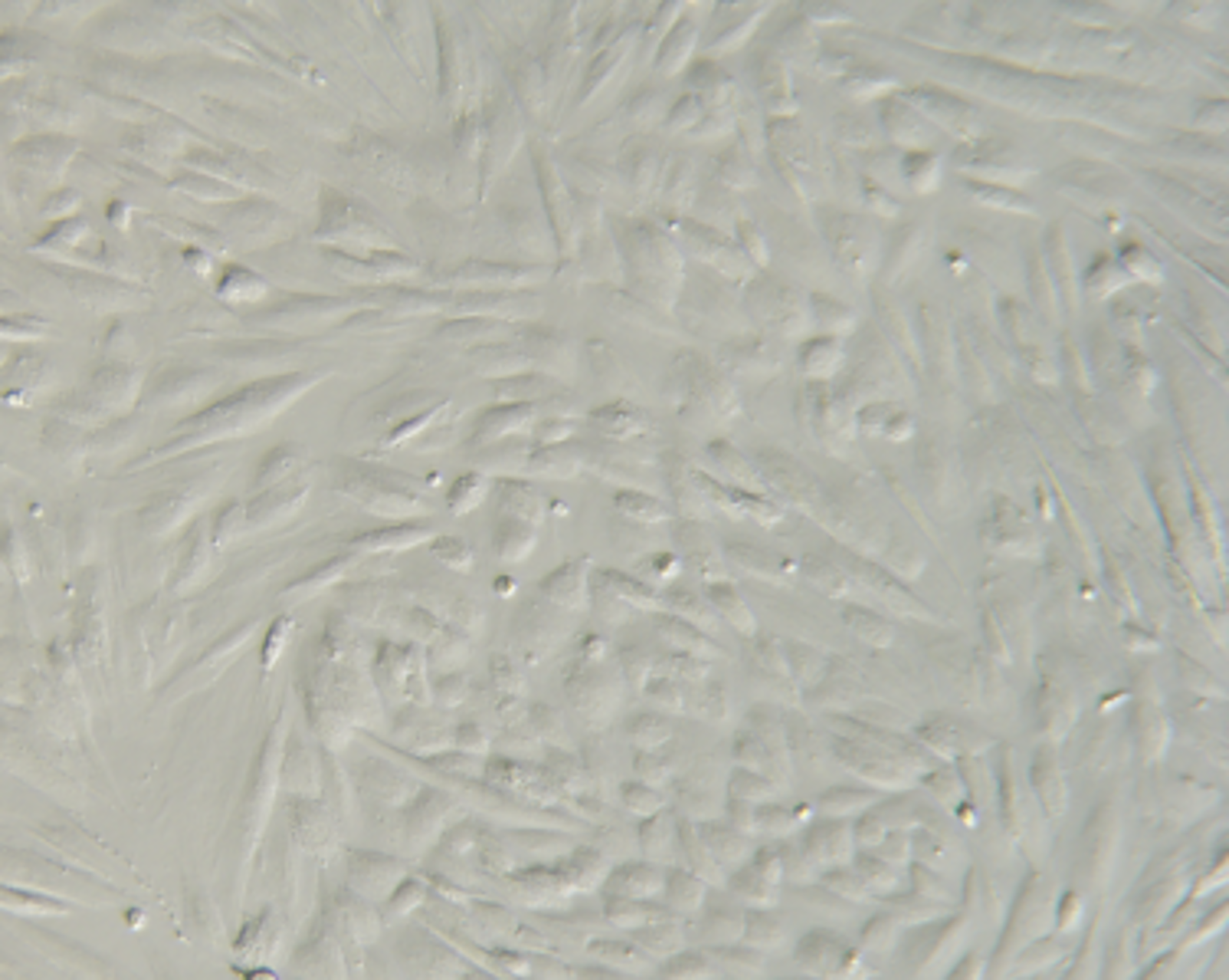

Supplement: Supplementary file 17 [file Image_15.png]
